# Design, Synthesis, and Cytotoxic Evaluation of *N*-Phenyl-3-(Thiophen-2-Yl)acrylamide Derivatives

**DOI:** 10.3390/molecules31183147

**Published:** 2026-09-08

**Authors:** Pablo S. Cavagnero, Felipe A. Zúñiga, Yaritza Ormazábal, Yeray A. Rodríguez-Núñez, David Ramírez, Paulina V. Hormazabal, Jorge Saavedra-Olavarría, Claudio A. Jiménez, Efraín Polo-Cuadrado, Jhon J. López

**Affiliations:** 1 Departamento de Química Orgánica, Facultad de Ciencias Químicas, Universidad de Concepción, Concepción 4130000, Chile; pcavagnero@udec.cl; 2 Departamento de Bioquímica Clínica e Inmunología, Facultad de Farmacia, Universidad de Concepción, Concepción 4130000, Chile; fzuniga@udec.cl (F.A.Z.); yormazabal@udec.cl (Y.O.); 3 Laboratorio de Síntesis y Reactividad de Compuestos Orgánicos, Departamento de Ciencias Químicas, Facultad de Ciencias Exactas, Universidad Andrés Bello, Santiago 8370146, Chile; yeray.rodriguez@unab.cl; 4 Departamento de Farmacología, Facultad de Ciencias Biológicas, Universidad de Concepción, Concepción 4130000, Chile; dramirezs@udec.cl (D.R.); paulinvalenzuela@udec.cl (P.V.H.); 5 Departamento de Química Orgánica, Facultad de Química y de Farmacia, Escuela de Química, Pontificia Universidad Católica de Chile, Santiago 7820436, Chile; jorge.saavedra@uc.cl

**Keywords:** acrylamide derivatives, thiophene scaffold, structure–activity relationship, cytotoxicity assay, drug-like properties

## Abstract

A series of *N*-phenyl-3-(thiophen-2-yl)acrylamide derivatives were designed and synthesized to investigate the influence of electronic and steric modifications on cytotoxic activity. Although related thiophene-containing acrylamides have been reported, the specific compounds studied in this study have not been previously described. Structural variations were introduced at the para position of the *N*-phenyl ring (H, CF_3_, and OCF_3_) and at the 5-position of the thiophene ring (H and CH_3_) to enable preliminary structure–activity relationship (SAR) analysis. The target compounds were synthesized via Knoevenagel–Doebner condensation, followed by carbodiimide-mediated amide coupling. The compounds were characterized using nuclear magnetic resonance (NMR) spectroscopy, high-resolution mass spectrometry (HRMS), Fourier-transform infrared (FTIR) spectroscopy, and high-performance liquid chromatography (HPLC). Computational evaluation indicated compliance with Lipinski’s rule of five and the absence of predicted mutagenic, tumorigenic, irritant, and reproductive risks. Cytotoxic activity was determined using the sulforhodamine B (SRB) assay against human breast adenocarcinoma (MCF-7), gastric adenocarcinoma (AGS), colorectal adenocarcinoma (Caco-2), oral squamous cell carcinoma (HSC-3), hepatocellular carcinoma (HepG2) cell lines and non-cancerous human dermal fibroblasts (HDF). Compounds **11** and **16** displayed the most consistent activity, with half-maximal inhibitory concentration (IC_50_) values of 379, 420, and 265 μM and 272, 256, and 242 μM against AGS, Caco-2, and MCF-7 cells, respectively. MCF-7 cells were the most sensitive model. The most active derivative was compound **16**, suggesting that the combination of a 5-methylthiophene moiety and a para-CF_3_ substituent may be beneficial within this limited compound series. These findings provide a preliminary evaluation of this scaffold and will help guide future studies on structural modifications.

## 1. Introduction

**Figure 1:**
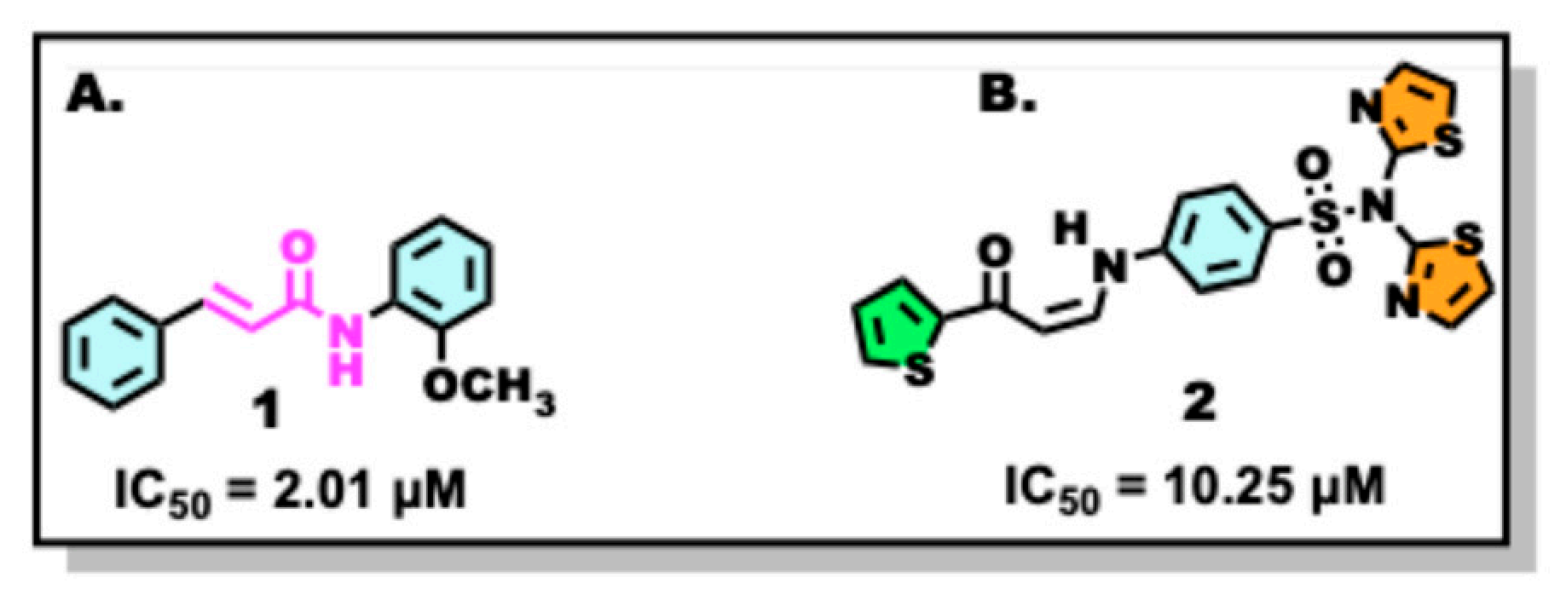
Chemical structures of compounds with reported anticancer activity. (**A**) *N*-(2-methoxyphenyl)cinnamide; (**B**) (*Z*)-4-(3-oxo-3-(thiophen-2-yl)prop-1-enylamino)-*N*-(thiazol-2-yl)benzenesulfonamide [7,9].

Cancer is the second leading cause of death worldwide. Cancer is a disease that originates in almost any organ or tissue and is characterized by the abnormal and uncontrolled growth of cells, which can invade various organs of the body (metastasis) [1]. One of the common characteristics of most cancers is the ability to evade natural cell death and achieve immortality [2,3]. In the past three decades, the number of patients diagnosed with tumors has more than doubled, and it is anticipated that by 2030, approximately 27 million individuals worldwide will be living with cancer, with 17 million deaths occurring annually [4]. Among the various types of cancer, breast cancer is the most common type of tumor worldwide. It is the leading cause of death in women [5]. Breast cancer is a multifactorial disease with relevant genetic, environmental, and lifestyle components [6]. Owing to its high mortality and prevalence, the search for new, effective, and low-toxicity anticancer agents remains a priority. In this context, *N*-phenyl-3-(phenyl)acrylamides have attracted attention because of their potent activity against a range of human cancer cell lines [7,8,9,10,11,12]. These compounds are present in many natural products and exhibit potent activities against various human cancer cell lines [7,8]. They can be synthesized via the reaction of acrylic acid derivatives with amines in the presence of a coupling reagent, such as 1-ethyl-3-(3-dimethylaminopropyl) carbodiimide hydrochloride (EDC) or N, N′-dicyclohexylcarbodiimide (DCC), and an appropriate catalyst [10]. For example, compound **1** (Figure 1A) has demonstrated significant antiproliferative activity in the human cell line MCF-7 [11].

Other interesting compounds containing five-membered aromatic thiophene heterocycles are notable for their anticancer, antibacterial, anti-inflammatory, and antiviral activities [4,13,14,15]. The thiophene ring provides synthetically accessible modification sites and acts as an important pharmacophore that can replace existing functionalities in drug candidates, making it a valuable fragment in medicinal chemistry [4,16]. Several commercially available drugs, including tipepidine, tiquizium bromide, dorzolamide, and sertaconazole nitrate, contain thiophene heterocycles, further highlighting their pharmacological relevance [13]. Thiophene derivatives have also demonstrated cytotoxic activity against cell lines MCF-7, with some compounds showing improved potency relative to doxorubicin [14], as illustrated by compound **2** (Figure 1B).

Considering the individual anticancer properties of acrylamides and thiophene derivatives, the combination of these two scaffolds in a single molecule may enhance cytotoxic activity and provide a useful template for further medicinal chemistry optimization. Structural modifications of the *N*-phenyl ring and thiophene moiety can modulate the electronic, steric, and lipophilic properties of the compound, potentially improving its potency, selectivity, and pharmacokinetic behavior. Previous reports indicate that acrylamide derivatives often exhibit IC_50_ values in the millimolar range [17], which, although moderate, are biologically relevant and can serve as a foundation for further chemical optimization.

**Figure 2:**
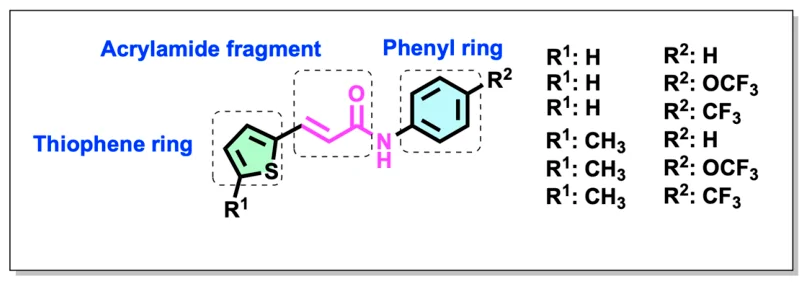
Chemical structures of the synthesized *N*-phenyl-3-(thiophen-2-yl)acrylamide derivatives.

Based on the activities of the reported acrylamides, this study aimed to synthesize and evaluate a series of *N*-phenyl-3-(thiophen-2-yl)acrylamide derivatives and examine their preliminary SAR (Figure 2). For this purpose, several substituents reported in anticancer compounds were selected to modify the *N*-phenyl ring, such as –H and –OCF_3_ or CF_3_ [12]. The unsubstituted phenyl derivative (H) was included as a reference structure to establish the baseline biological activity of the scaffold. The para-CF_3_ and para-OCF_3_ substituents were selected because fluorinated groups are frequently employed in medicinal chemistry to modulate lipophilicity, metabolic stability, membrane permeability, and target interactions. Moreover, CF_3_ is a strong electron-withdrawing group, whereas OCF_3_ combines electron-withdrawing properties with increased steric bulk and lipophilicity. Therefore, substituents were incorporated to explore how electronic and steric effects influenced the cytotoxic activity of the target compounds. We also substituted the thiophene ring at the 5-position with a methyl group to assess its effect on activity. The synthetic strategy and evaluation of the anticancer activity against cancer cell lines are described herein.

Despite the growing interest in acrylamide- and thiophene-based compounds as potential anticancer agents, there is limited information on the cytotoxic activity of *N*-phenyl-3-(thiophen-2-yl)acrylamide derivatives and the influence of systematic structural modifications on their biological behavior. In particular, no study has evaluated the combined effect of para-substitution on the *N*-phenyl ring and substitution at the 5-position of the thiophene ring within this scaffold using a comparative structure–activity relationship approach. Addressing this knowledge gap may provide valuable insights into the rational design of improved acrylamide-based anticancer agents. According to the available literature and SciFinder database records, compounds **11**–**14** are listed as commercially available substances without associated literature reports, whereas compounds **15** and **16** yielded no records in the database. Therefore, this appears to be the first report describing the synthesis, characterization, and cytotoxic evaluation of compounds **11**–**16**. Accordingly, the present study focuses on the preparation of these previously undescribed derivatives and on evaluating how structural modifications within the *N*-phenyl-3-(thiophen-2-yl)acrylamide framework influence their cytotoxic activity and preliminary structure–activity relationships.

## 2. Results and Discussion

### 2.1. Design

The *N*-phenyl-3-(thiophen-2-yl)acrylamide derivatives were designed by combining an acrylamide framework and thiophene moiety, both of which have been associated with anticancer activity in previous studies. The phenyl ring was incorporated as a versatile aromatic platform that enabled systematic structural modification through para-substitution (Figure 2). Structural variations were introduced at two key positions, the fourth position of the phenyl ring (–H, –CF_3_, –OCF_3_) and the fifth position of the thiophene ring (–H, –CH_3_), to investigate the steric, electronic, and lipophilic effects of these groups on biological activity. The H substituent was used as a reference group, whereas CF_3_ and OCF_3_ were selected as medicinally relevant fluorinated groups to systematically probe the influence of electronic withdrawal, lipophilicity, and steric effects on the biological properties of the target scaffold [12].

### 2.2. In Silico Prediction of Drug-like and Toxicological Properties

Before experimental synthesis, these derivatives were evaluated in silico to assess their drug-like properties and toxicity (Table 1). The in silico evaluation of the *N*-phenyl-3-(thiophen-2-yl)acrylamide derivatives confirmed that the entire series complied with Lipinski’s rule of five [17,18] with molecular weights ≤ 500 Da, moderate lipophilicity (iLogP 2.33–2.99), and acceptable ranges of hydrogen bond donors (HBD = 1) and acceptors (HBA = 1–5). These values, together with the low calculated topological polar surface area (TPSA = 57.34–66.57 Å^2^), suggest adequate passive membrane permeability and potential oral bioavailability of these compounds in the human body. The moderate number of rotatable bonds (4–6) suggests that the compounds maintain a balance between structural flexibility and conformational stability, which may favor target binding without compromising the pharmacokinetic properties.

Importantly, toxicity predictions did not reveal MUT, IRT, TUM, or REP risks for any of the tested derivatives [19], further supporting the safety of the scaffold at the preliminary in silico level (Table 1). This combination of favorable physicochemical parameters and the absence of predicted toxicological alerts reinforces the drug-like potential of acrylamide–thiophene derivatives and justifies their synthesis and subsequent biological evaluation.

**Table 1:** Predicted drug-like properties of the compounds calculated using two online servers, SwissADME [20] and DataWarrior [21].

Compounds	MW (g/mol)	iLogP	HBD	HBA	TPSA (${Å}^{2}$)	Heavy Atoms	Rotatable Bonds	MUT	IRT	TUM	REP
**11**	229.30	2.33	1	1	57.34	16	4	None	None	None	None
**12**	313.29	2.75	1	5	66.57	21	6	None	None	None	None
**13**	297.30	2.80	1	4	57.34	11	5	None	None	None	None
**14**	243.32	2.57	1	1	57.34	17	4	None	None	None	None
**15**	327.32	2.99	1	5	66.57	22	6	None	None	None	None
**16**	311.32	2.91	1	4	57.34	21	5	None	None	None	None

MW, molecular weight; iLogP, partition coefficient; HBD, hydrogen bond donor; HBA, hydrogen bond acceptor; TPSA, topological polar surface area; MUT, mutagenic effects; IRT, irritant effects; TUM, tumorigenic effects; REP, reproductive effects.

These in silico predictions provide important guidance for selecting target compounds for synthesis, ensuring that the designed derivatives possess favorable physicochemical characteristics prior to chemical preparation. By integrating these predictive analyses, this study aimed to maximize the likelihood that the synthesized compounds would display adequate bioavailability and measurable cytotoxic activity, thereby linking rational design with practical experimentation.

### 2.3. Chemical Synthesis

**Scheme 1:**
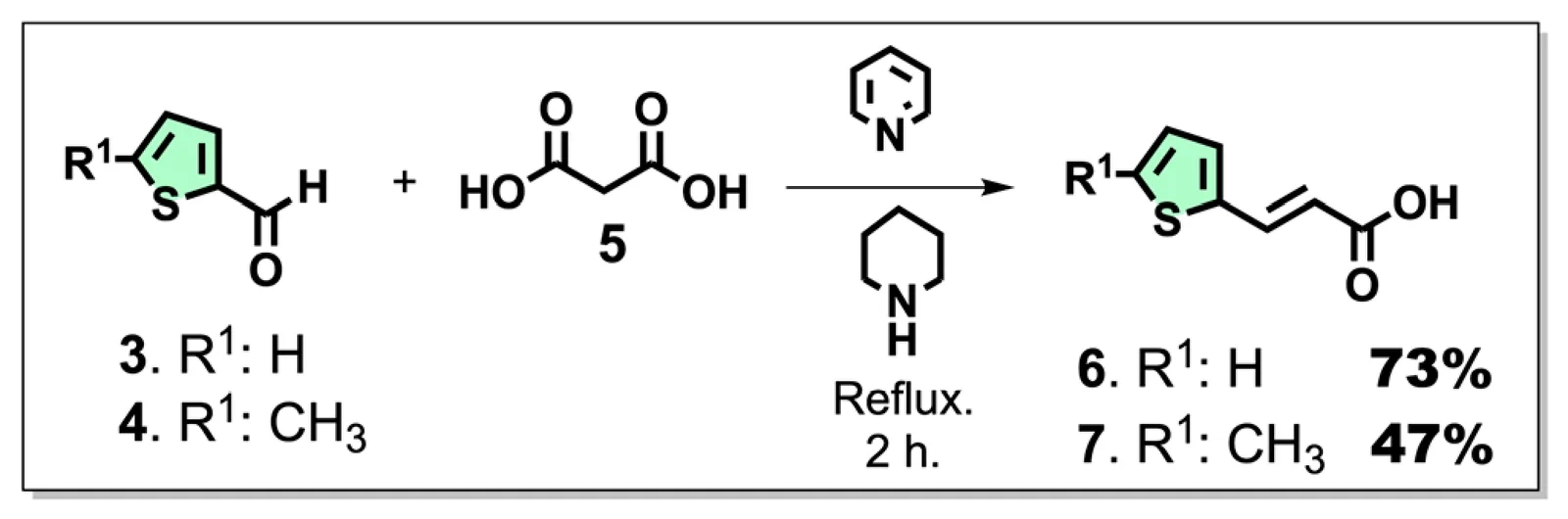
Chemical synthesis of acrylic acid derivatives **6** and **7**. Reagents and conditions: 2-thiophenocarbaldehyde (**3** or **4**; 4.5 mmol), malonic acid (**5**; 9 mmol), piperidine (45 μL), and pyridine (2.25 mL) were refluxed for 2 h.

Acrylic acid derivatives **6** and **7** were synthesized as depicted in Scheme 1. They were characterized by the presence or absence of a methyl group at the 5-position of the thiophene ring. To obtain these derivatives, a Knoevenagel–Doebner reaction was used, which consists of the reaction of different commercially available 2-thiophenocarbaldehydes (**3** and **4**) and malonic acid (**5**) using piperidine and pyridine [22,23].

**Scheme 2:**
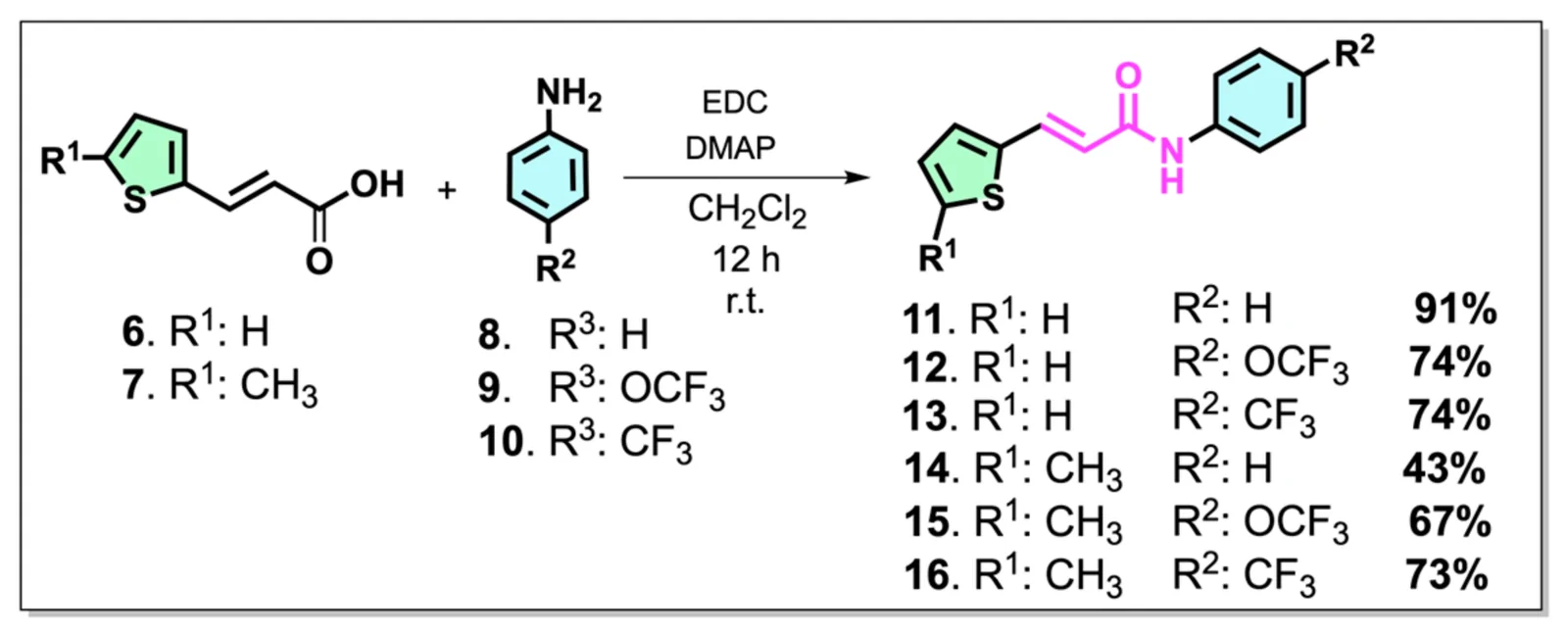
Chemical synthesis of *N*-phenyl-3-(thiophen-2-yl)acrylamide **11**–**16**. Reagents and conditions: acrylic acid derivatives (**6** or **7**; 1.1 mmol), aniline (**8**–**10**; 1 mmol), CH_2_Cl_2_ 5 mL, DMAP (3 mmol), and EDC (2 mmol) at room temperature for 12 h.

Subsequently, a variant of the Steglich reaction was used to obtain compounds **11**–**16** (Scheme 2) by reacting acrylic acid derivatives **6** and **7** with several substituted anilines in the para (**8**–**10**) position with fluorinated substituents such as -OCF_3_ and -CF_3_, using anhydrous CH_2_Cl_2_ as the solvent, DMAP (4-Dimethylaminopyridine) as the catalyst, and EDC as the coupling agent [24,25].

The progress of the reactions was monitored by thin-layer chromatography (TLC), and all ligands were purified by column chromatography. The structural characteristics of the new derivatives were determined using ^1^H and ^13^C-NMR spectroscopy and HRMS, and their melting points were determined using a melting point apparatus. In the FTIR spectra, characteristic absorptions corresponding to the amide N-H stretching vibration were observed at 3242–3256 cm^−1^, while the carbonyl group of the acrylamide moiety appeared at 1650–1664 cm^−1^. The presence of the conjugated α,β-unsaturated system was further supported by absorption bands at 1606–1624 cm^−1^ associated with the C=C stretching vibration. Compounds bearing trifluoromethyl or trifluoromethoxy substituents (**12**, **13**, **15**, and **16**) exhibited characteristic C-F stretching bands between 1108 and 1165 cm^−1^. The ^1^H NMR spectra were consistent with the proposed structures. All derivatives displayed a singlet at δ 10.1–10.5 ppm attributable to the amide NH proton. The two olefinic protons of the α,β-unsaturated amide system appeared as doublets with coupling constants of approximately 15.3–15.4 Hz, confirming the *E* configuration of the double bond. Signals corresponding to the thiophene and aromatic phenyl protons were observed within the expected aromatic region between δ = 6.8 and 7.9 ppm. Furthermore, compounds **14**–**16**, which contain a methyl-substituted thiophene ring, showed an additional singlet at δ 2.47–2.48 ppm corresponding to the CH_3_ group.

The ^13^C NMR spectra further supported the assigned structures, showing characteristic resonances for the amide carbonyl carbon at δ 163–164 ppm together with the expected aromatic, olefinic, and thiophene carbon signals. The molecular compositions were confirmed by HRMS, which showed excellent agreement between the calculated and experimental [M + H]^+^ values for all synthesized compounds.

The isolated yields of compounds **11**–**16** varied from moderate to excellent. Although no systematic mechanistic investigation has been performed, these differences may be attributed to the electronic and steric effects of the substituents present in the aniline precursors and thiophene framework. In particular, electron-withdrawing groups such as CF_3_ and OCF_3_ can affect the nucleophilicity of the aniline nitrogen during the EDC/DMAP-mediated amidation process, whereas substitution on the thiophene ring may influence both the reaction efficiency and product recovery during purification. Additionally, variations in the chromatographic isolation and handling of the different derivatives may have contributed to the observed differences in the isolated yields.

### 2.4. Cytotoxicity Assays

**Figure 3:**
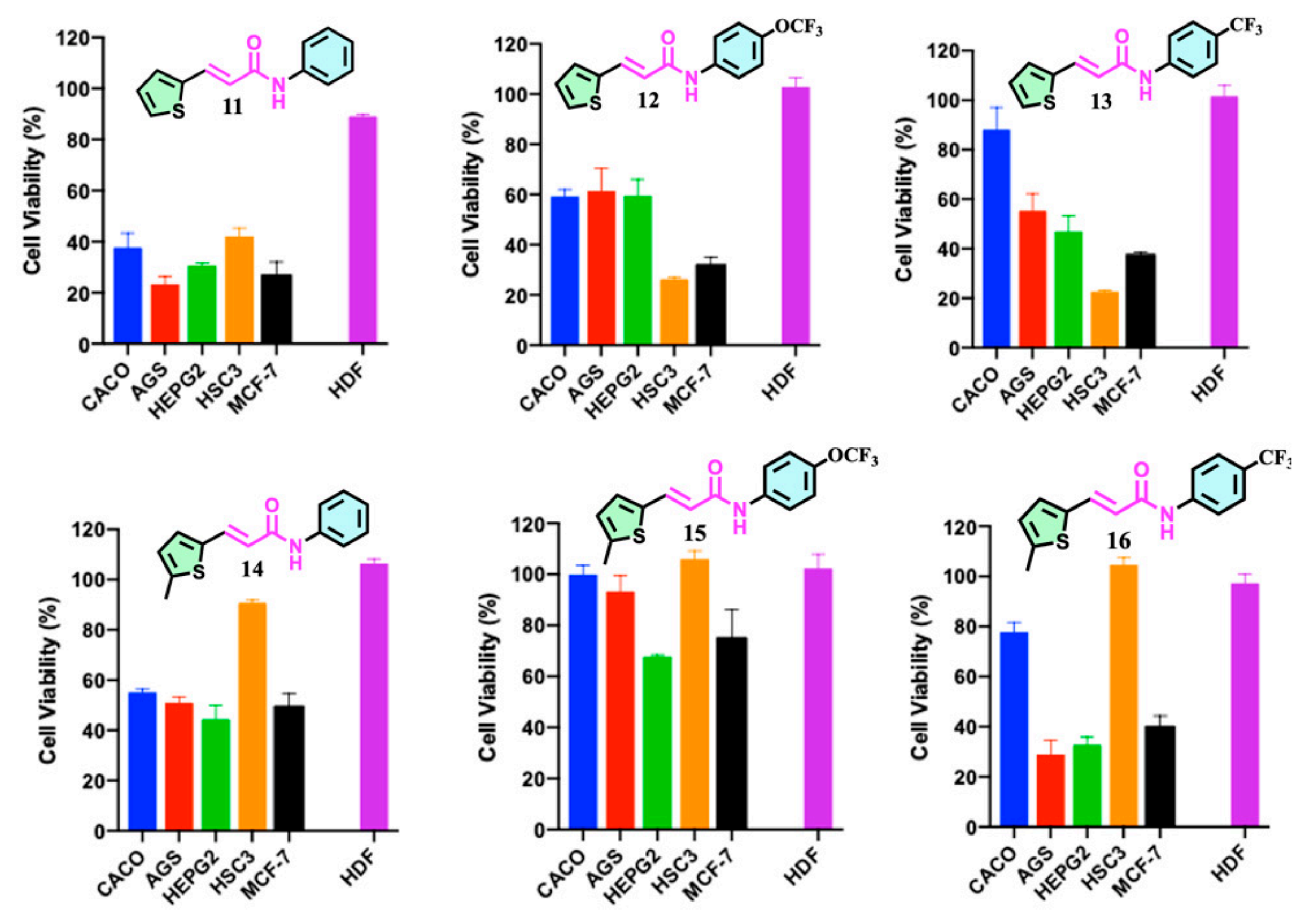
General cytotoxic screening of *N*-phenyl-3-(thiophen-2-yl)acrylamides (**11**–**16**). Cell viability (%) was determined after treating AGS, Caco-2, HepG2, MCF-7, HSC-3, and non-tumoral HDF cells with each compound at the highest concentration tested within the dose–response range. Data are expressed as the percentage of viable cells relative to untreated controls.

**Figure 4:**
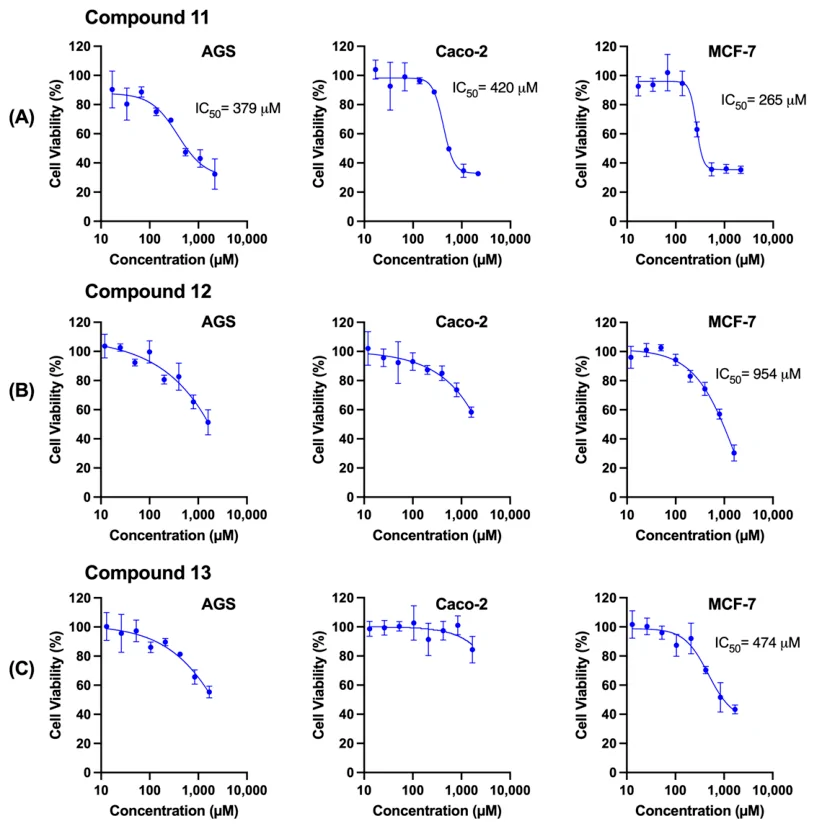
Dose–response curves and IC_50_ determination for compounds **11** (**A**), **12** (**B**), and **13** (**C**). AGS, Caco-2, and MCF-7 cells were exposed to increasing concentrations of compounds **11**, **12**, and **13**, and cell viability (%) was quantified relative to that of the untreated controls. Sigmoidal nonlinear regression was used to estimate the IC_50_ values when 50% inhibition was achieved within the tested range; when 50% inhibition was not reached, the IC_50_ was not determined.

**Figure 5:**
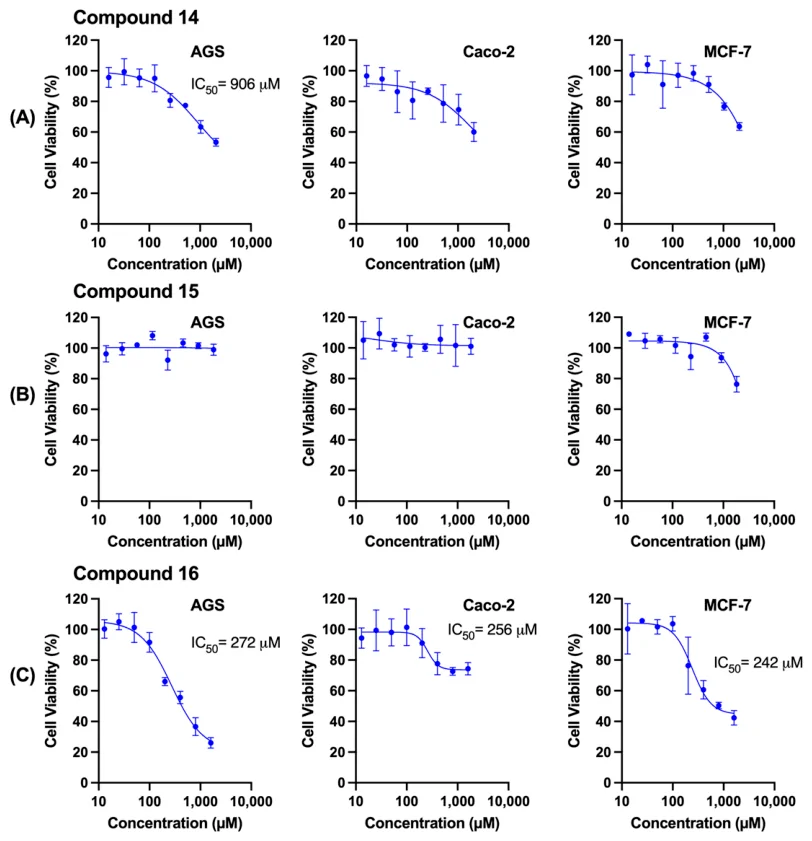
Dose–response curves and IC_50_ determination for compounds **14** (**A**), **15** (**B**), and **16** (**C**). AGS, Caco-2, and MCF-7 cells were treated with increasing concentrations (10–10,000 µM) of compounds **14**, **15**, and **16**, and cell viability (%) was quantified relative to that of the untreated controls. The IC_50_ values were calculated by nonlinear regression only when 50% inhibition was reached; otherwise, the IC_50_ could not be reliably determined.

The cytotoxic activities of the synthesized compounds (**11**–**16**) were initially evaluated in a panel of human cancer cell lines, including Caco-2, AGS, HepG2, HSC-3, and MCF-7, at a fixed concentration of 100 µM as a preliminary screening concentration (Figure 3). Compounds showing measurable antiproliferative effects were subsequently evaluated in dose–response experiments using a broader concentration range (10–10,000 µM) to determine their IC_50_ values (Figure 4 and Figure 5). This initial screening approach allowed for a rapid comparison of the biological effects of the tested compounds under uniform experimental conditions and facilitated the selection of derivatives for further quantitative evaluation. HDF were included as a non-tumor cell model to assess compound selectivity. Cell viability was determined after 24 h of incubation using the SRB assay (Figure 3) [26].

As shown in Figure 3 (general screening), marked differences in cell viability were observed among the synthesized derivatives, highlighting a strong dependence on both the substitution pattern and cellular context. Compounds **11** and **16** consistently exhibited the greatest antiproliferative effects across several tumor models, particularly in AGS and MCF-7 cells, while maintaining high viability in non-tumoral HDFs, suggesting selectivity toward cancer cells.

In contrast, compounds **12**–**14** displayed intermediate and cell line-dependent activities, whereas compound **15** was inactive under the experimental conditions. Overall, these findings indicate that subtle structural modifications within the acrylamide–thiophene scaffold strongly influence biological activity and support further dose–response evaluation of the most active derivatives in this series.

These initial cytotoxicity profiles demonstrated that the biological activity of this series was influenced by both molecular structure and cancer cell type. The preferential reduction in the viability of MCF-7 and AGS cells, combined with the minimal effects observed in non-cancerous fibroblasts, suggests selective cytotoxic behavior. This provided a solid foundation for the subsequent determination of the IC_50_ values and preliminary SAR analysis, which are discussed in the following sections.

### 2.5. Dose–Response Analysis and IC_50_ Determination

Following preliminary screening, dose–response studies were performed on AGS, Caco-2, and MCF-7 cells to characterize the antiproliferative effects of the synthesized compounds. AGS, Caco-2, and MCF-7 cells were selected because they provided reproducible and representative responses encompassing distinct sensitivity profiles, thereby enabling a robust quantitative comparison of the cytotoxic potency across the compounds. Nonlinear regression analysis was used to determine the IC_50_ values for the most active compounds (Figure 4 and Figure 5). Among the series, only compounds **11** and **16** produced clear sigmoidal dose–response curves in all three cell lines, allowing reliable IC_50_ determination. Compound **11** exhibited a clear, dose-dependent cytotoxic effect, with IC_50_ values of 379 µM in AGS, 420 µM in Caco-2, and 265 µM in MCF-7 cells, respectively (Figure 4A). MCF-7 cells were the most sensitive, whereas Caco-2 cells required slightly higher concentrations to achieve 50% cell viability inhibition. The curves displayed a classical sigmoidal profile with a progressive reduction in viability at concentrations > 100 µM. Compound **16** exhibited the most pronounced cytotoxic profile among the methylated thiophene derivatives, with well-defined sigmoidal curves and IC_50_ values of 272 µM in AGS, 256 µM in Caco-2, and 242 µM in MCF-7 cells (Figure 5C), demonstrating comparable potency across the three tumor models, with slightly greater activity in MCF-7 cells.

In contrast, compounds **12**–**15** exhibited limited or no cytotoxic activity within the tested concentration range. Compound **12** displayed differential behavior across cell lines: in AGS cells, viability decreased progressively, but 50% inhibition was not clearly reached, preventing robust IC_50_ calculation; in Caco-2 cells, the reduction was modest and did not approach 50%; in contrast, MCF-7 cells showed a well-defined sigmoidal curve, yielding an IC_50_ of 954 µM, indicating that compound **12** exhibits weak but measurable activity specifically in the breast cancer model, with a strong dependence on cellular context. Compound **13** exhibited limited activity in AGS and Caco-2 cells, with viability not decreasing below 50% across all tested concentrations. However, in MCF-7 cells, a clear dose-dependent inhibition was observed, with an IC_50_ of 474 µM, suggesting a cell line-specific sensitivity of the breast cancer model to this derivative. Compound **14** achieved 50% inhibition only in AGS cells, with an IC_50_ of 906 µM. In Caco-2 and MCF-7 cells, the reduction in viability was moderate and did not reach the 50% threshold, preventing IC_50_ determination in those models. Compound **15** did not produce a significant dose-dependent decrease in viability across any of the three cell lines; cell viability remained close to the control levels throughout the concentration range, indicating a lack of relevant antiproliferative activity and precludin_g_ the IC_50_ calculation.

Compared with the reference drug doxorubicin (Table 2), compounds **11** and **16** exhibited substantially higher IC_50_ values (242–420 µM vs. 0.38–0.52 µM for doxorubicin), indicating a relatively weak antiproliferative activity. Nevertheless, both compounds displayed markedly lower toxicity toward non-cancerous HDF (IC_50_ > 1000 µM) than doxorubicin (IC_50_ = 2.8 µM), suggesting a degree of selectivity toward cancer cells under the tested conditions.

Overall, the IC_50_ analysis identified compounds **11** and **16** as the most active derivatives, whereas compounds **12**–**14** displayed weaker and cell line-dependent effects, and compound **15** remained essentially inactive. An overall cell line-dependent sensitivity trend was observed, with MCF-7 being the most sensitive cell line, followed by AGS and Caco-2 cell lines. However, compound **16** exhibited a slightly different profile (MCF-7 > Caco-2 > AGS), indicating that this trend was not strictly conserved across all derivatives.

Notably, structurally simple acrylamides or those with a lower degree of conjugation have shown IC_50_ values in the micromolar-to-millimolar range in Caco-2 and A549 cells [26,27,28]. In this context, the IC_50_ values determined for compounds **11** and **16** indicate weak but measurable cytotoxicity. Although the potency remains substantially lower than that of clinically used anticancer agents, these results provide initial information regarding the biological behavior of this compound class and may assist future optimization efforts.

Interestingly, MCF-7 cells consistently displayed greater sensitivity to multiple derivatives (**11**, **12**, **13**, and **16**), suggesting that this scaffold preferentially affects breast cancer cells. This behavior may be associated with intrinsic differences in cellular metabolism, membrane composition, redox homeostasis, and susceptibility to electrophilic stress. However, additional mechanistic studies are necessary to elucidate the underlying molecular basis of this observation. Second, the introduction of a 5-methyl group on the thiophene ring combined with a para-CF_3_ substituent (**16**) resulted in a marked improvement in potency compared to its non-methylated analog **13**, which exhibited only moderate activity in MCF-7 cells.

### 2.6. Preliminary (SAR) Analysis

The cytotoxic data obtained for compounds **11**–**16** indicate that modest structural modifications within the acrylamide–thiophene framework resulted in measurable differences in the biological response. However, it is important to acknowledge that with only six compounds and minimal structural variations, our analysis can only identify preliminary trends rather than establishing definitive structure–activity relationships (SARs). The structural modifications in this series were limited to two positions: the para position of the *N*-phenyl ring (–H, –CF_3_, –OCF_3_) and the 5-position of the thiophene ring (–H, –CH_3_). Although these variations allow pairwise comparisons, the limited number of derivatives and the absence of other substitution patterns prevent the establishment of a robust SAR. Consequently, the following discussion should be interpreted as a preliminary trend analysis intended to guide future optimization efforts, rather than as a definitive structure–activity model. Accordingly, the observations discussed below should be considered hypothesis-generating and intended to support future optimization efforts rather than to establish definitive SAR conclusions.

A matched-pair analysis of non-methylated and methylated thiophene analogs (**11**/**14**, **12**/**15**, and **13**/**16**) revealed that methyl incorporation did not systematically enhance cytotoxic potency. For the –H phenyl-substituted pair, the methylated analog **14** showed reduced activity compared to **11**, and for the –OCF_3_ phenyl-substituted pair, compound **15** exhibited diminished potency relative to **12**. In contrast, a clear improvement was observed only for the –CF_3_ phenyl-substituted pair, where compound **16** exhibited substantially lower IC_50_ values and a broader activity profile than its non-methylated counterpart **13**, extending its antiproliferative effects to AGS and Caco-2 cells. This inconsistent pattern indicates that the effect of the 5-methyl substituent is highly context-dependent and emerges only when combined with specific electronic features of the *N*-phenyl ring, particularly that of the para-CF_3_ group. For the –H and –OCF_3_ phenyl-substituted analogs, methyl introduction was either neutral or detrimental to activity, suggesting that the beneficial contribution of the 5-methyl group is not generalizable across this series.

The effect of the para-phenyl substituent varied depending on the substitution state of thiophene. Among the non-methylated thiophene series, the activity trend observed in MCF-7 cells was as follows: –H (**11**, IC_50_ = 265 µM) > –CF_3_ (**13**, IC_50_ = 474 µM) > –OCF_3_ (**2**, IC_50_ = 954 µM). These results indicate that the introduction of a para-CF_3_ substituent alone did not improve the activity relative to the unsubstituted analog. However, this order changed markedly in the methylated thiophene series, –CF_3_ (**16**, IC_50_ = 242 µM) > –H (**14**, IC_50_ > 1000 µM) > –OCF_3_ (**15**, IC_50_ > 1000 µM), where the –CF_3_ group was essential for maintaining measurable activity. These observations suggest that the effect of the para-phenyl substituent is not additive but rather synergistic with the thiophene substitution pattern and that optimal activity within this series requires a favorable combination of both structural features rather than the presence of any single substituent alone. The para-OCF_3_ derivatives (**12** and **15**) consistently displayed reduced potency, which may be attributed to the greater steric bulk and additional conformational flexibility introduced by this group compared to –CF_3_, potentially affecting the molecular planarity and conjugation efficiency within the extended π-system. However, this hypothesis requires further validation in larger studies.

Compound **16** exhibited the highest activity in the study series, which may be associated with the cooperative interplay between hydrophobic and electronic effects. The electron-withdrawing –CF_3_ group may enhance the electrophilic character of the α,β-unsaturated amide system, potentially facilitating its interaction with intracellular nucleophilic targets such as cysteine residues. The 5-methyl group on the thiophene ring may improve lipophilic interactions and membrane permeability. However, this hypothesis remains speculative and requires further experimental validation through mechanistic studies, including target identification, cellular uptake assays, and reactivity studies using model nucleophiles. The presence of the α,β-unsaturated amide moiety may also contribute to the observed biological effects through interactions with intracellular nucleophilic biomolecules; however, the precise molecular targets remain unknown, and this hypothesis requires further investigation.

Based on these preliminary trends, several directions can be proposed for future SAR optimization, including the expansion of the compound library to include additional substituents at the para position (e.g., –F, –Cl, –OMe, –CN, and–NO_2_) to better define the electronic requirements for activity, exploration of meta- and ortho-substitution patterns on the *N*-phenyl ring, variation in the thiophene substitution pattern (e.g., 3-methyl, 4-methyl, or dimethyl derivatives), modification of the acrylamide linker to assess the role of the Michael acceptor moiety, and mechanistic studies to identify the molecular targets and pathways involved. In summary, this study provides valuable preliminary insights into the structural features that influence the cytotoxic activity of *N*-phenyl-3-(thiophen-2-yl)acrylamide derivatives. However, the limited compound library size and minimal structural diversity hinder the establishment of a definitive SAR. The observed trends, particularly the context-dependent effect of the 5-methyl substituent and the apparent synergy between the para-CF_3_ and thiophene-methyl groups, should be considered hypothesis-generating and will require validation and further elaboration through expanded structure–activity relationship studies.

## 3. Materials and Methods

### 3.1. Chemical Synthesis

For the chemical synthesis of all compounds, commercial reagents and solvents were used (Sigma-Aldrich (St. Louis, MO, USA), MERCK (Darmstadt, Germany), and AmBeed (Arlington Heights, IL, USA)) employing previously described synthetic methodologies [22,23,24,25]. Pre-coated silica gel 60 plates (Merck 60 F254 0.2 mm; Merck KGaA, Darmstadt, Germany) were used for TLC, and silica gel 60 (0.015–0.040 mm) was used for column chromatography. Spots on thin-layer chromatograms were visualized by spraying with Dragendorff’s reagent or by exposure to ultraviolet light at a wavelength of 254 nm. ^1^H and ^13^C NMR spectra were recorded at 400 and 100 MHz for ^1^H and ^13^C, respectively, on Bruker Avance 400 spectrometers. Chemical shifts are reported as δ values (ppm) relative to an internal standard of tetramethylsilane (TMS) in deuterated chloroform (CDCl_3_) and deuterated dimethyl sulfoxide (DMSO-*d*_6_), and the coupling constants (*J*) are expressed in Hz. HRMS spectra were recorded using a Bruker Compact QqTOF spectrometer (Billerica, MA, USA). The analysis was performed via direct injection. A Hamilton syringe (500 μL, Reno, NV, USA) was used at a flow rate of 2 μL/min with an InfusionONE Syringe Pump NE-300 (New Era Pump Systems, Inc., Farmingdale, NY, USA). An electrospray ionization (ESI) source with a voltage of 4500 V, gas temperature of 180 °C, flow rate of 4 L/min, and nebulizer at 0.4 bar was used for analysis. The purity of the final compounds was determined using HPLC LC20AT Shimadzu (Nakagyo-ku, Japan). FTIR spectra were obtained using a NEXUS Nicolet Magna 550 FT-IR instrument (Madison, WI, USA). The results are provided in Supplementary Information. Melting points (m.p.) were determined using a Stuart SMP10 apparatus (Bibby Sterlin Ltd., Staffordshire, UK) and were not corrected.

### 3.2. General Procedure for the Preparation of Acrylic Acid Derivatives 6 and 7 via the Knoevenagel–Doebner Reaction

A round-bottom flask (50 mL) was charged with 4.5 mmol (505 mg) of a 2-thiophenecarbaldehyde derivative (**3** or **4**), 9 mmol (937 mg) of malonic acid (**5**), 2.25 mL pyridine, and 45 μL piperidine. The reaction mixture was stirred and refluxed for 2 h. The progress of the reaction was monitored using thin-layer chromatography (TLC). The mixture was allowed to cool to room temperature and then to a water-ice bath, to which 20 mL of distilled water and 10 mL of HCl solution (37%) were added. The formed solid was separated by vacuum filtration and purified by crystallization using 20 mL of a water-ethanol solution (1:1). The formed crystals were filtered under vacuum and allowed to dry at room temperature to obtain the corresponding compounds.

*(E)-3-(thiophen-2-yl)acrylic acid* (**6**). The compound formed brown crystals. (508 mg, 73%). ^1^H NMR (400 MHz, CDCl_3_) δ: 7.75 (d, *J* = 17.1 Hz, 1H), 7.22 (m, 3H), 6.17 (d, *J* = 17.0 Hz, 1H). ^13^C NMR (101 MHz, CDCl_3_) δ: 169.13, 139.38, 137.87, 130.94, 128.57, 128.02, 116.74. FTIR (cm^−1^): 3200–2500 (O-H), 1691 (C=O), 1610 (C=C), 1264 (C-O). m.p. 147–149 °C.

(*E)-3-(5-methylthiophen-2-yl)acrylic acid* (**7**). The compound formed yellow crystals (356 mg, 47%). ^1^H NMR (400 MHz, CDCl_3_) δ: 7.93 (d, *J* = 15.6 Hz, 1H), 7.23 (d, *J* = 3.5 Hz, 1H), 6.87 (d, *J* = 2.4 Hz, 1H), 6.23 (d, *J* = 15.6 Hz, 1H), 2.65 (s, 3H). ^13^C NMR (101 MHz, CDCl_3_) δ: 172.54, 145.07, 139.76, 137.25, 132.49, 126.70, 114.42, 15.88. FTIR (cm^−1^): 3200–2500 (O-H), 2600 (C-H, sp3), 1709 (C=O), 1614 (C=C), 1208 (C-O). m.p. 164–166 °C.

### 3.3. General Procedure for the Preparation of N-phenyl-3-(thiophen-2-yl)acrylamide Derivatives 11–16 via the Steglich Reaction Variant

An acid derivative (**6** or **7**, 50 mL, 1.1 mmol) and 5 mL of anhydrous CH_2_Cl_2_ were added to a round-bottomed flask, followed by the addition of 2.0 mmol (383 mg) of EDC and 3.0 mmol (367 mg) of DMAP. The reaction mixture was cooled to 5 °C for 30 min, and aniline (**8**–**10**; 1 mmol in 5 mL of CH_2_Cl_2_) solution was added slowly. The mixture was then refluxed for 12 h under nitrogen while being stirred. The progress of the reaction was monitored using thin-layer chromatography (TLC). Next, 10 mL of CH_2_Cl_2_ was added, and extraction was performed using two 10 mL portions of 1.5 M HCl. The organic phase was separated and washed with 10 mL of 10% (*m*/*v*) NaOH solution and 10 mL of distilled water. Anhydrous Na_2_SO_4_ was then added, and the mixture was filtered and concentrated under reduced pressure. The product was purified by column chromatography using CH_2_Cl_2_ to obtain the corresponding compound.

*(E)-N-phenyl-3-(thiophen-2-yl)acrylamide* (**11**). The obtained compound was a yellow solid (209 mg, 91% yield). ^1^H NMR (400 MHz, DMSO-*d*_6_) δ: 10.18 (s, 1H), 7.75 (d, *J* = 15.4 Hz, 1H), 7.69–7.62 (m, 3H), 7.45 (d, *J* = 3.6 Hz, 1H), 7.32 (t, *J* = 7.8 Hz, 2H), 7.14 (dd, *J =* 5.1, 3.6 Hz, 1H), 7.06 (t, *J =* 7.4 Hz, 1H), 6.60 (d, *J* = 15.4 Hz, 1H). ^13^C NMR (101 MHz, DMSO-*d*_6_) δ: 163.28, 139.76, 139.29, 133.19, 131.33, 128.82, 128.44, 123.34, 120.88, 120.81, 119.13. FTIR (cm^−1^): 3256 (N-H), 3130 (C_sp2_-H vinylic), 3067 (C_sp2_-H aromatic), 1653 (C=O), 1615 (C=C vinylic), and 1543 (C=C aromatic). HRMS (ESI) *m*/*z*: 230.0634 [M + H]^+^ (calculated for C_13_H_11_NOS: 229.0561). HPLC purity = 99.8% (mobile phase: MeOH, 1 mL/min, tR = 2.357 min). m.p. 155–157 °C.

*(E)-3-(thiophen-2-yl)-N-(4-trifluoromethoxy)phenyl)acrylamide* (**12**). The obtained compound was a brown solid (232 mg, 74% yield). ^1^H NMR (400 MHz, DMSO-*d*_6_) δ: 10.39 (s, 1H), 7.84–7.75 (m, 3H), 7.66 (d, *J* = 5.1 Hz, 1H), 7.45 (d, *J* = 3.0 Hz, 1H), 7.33 (d, *J* = 8.2 Hz, 2H), 7.16–7.11 (m, 1H), 6.58 (d, *J* = 15.4 Hz, 1H). ^13^C NMR (101 MHz, DMSO-*d*_6_) δ: 163.49, 143.61, 139.69, 138.55, 133.68, 131.60, 128.66, 128.53, 121.77, 121.48, 120.48, 120.45, 120.38, 120.36, 120.21 (q ^1^*J*_CF_ = 252.6 Hz). FTIR (cm^−1^): 3256 (N-H), 3130 (C_sp2_-H vinylic), 3067 (C_sp2_-H aromatic), 1663 (C=O), 1624 (C=C vinylic), 1523 (C=C aromatic), 1204 (C-O), and 1153 (C-F). HRMS (ESI) *m*/*z*: 314.0470 [M + H]^+^ (calculated for C_14_H_10_F_3_NO_2_S: 313.0384). HPLC purity = 99.9% (mobile phase: MeOH, 1 mL/min, tR = 2.371 min). m.p. 168–170 °C.

*(E)-3-(thiophen-2-yl)-N-(4-trifluoromethyl)phenyl)acrylamide* (**13**). The compound was obtained as a brown solid (232 mg, 74% yield). ^1^H NMR (400 MHz, DMSO-*d*_6_) δ: 10.52 (s, 1H), 7.89 (d, *J* = 8.7 Hz, 2H), 7.80 (d, *J* = 15.4 Hz, 1H), 7.69 (d, *J* = 9.0 Hz, 3H), 7.48 (d, *J* = 3.7 Hz, 1H), 7.17–7.11 (m, 1H), 6.59 (d, *J* = 15.4 Hz, 1H). ^13^C NMR (101 MHz, DMSO-*d*_6_) δ: 163.79, 142.83, 139.56, 134.10, 131.81, 128.86, 128.56, 126.15 (q ^3^*J*_CF_ = 3.70 Hz), 123.26 (q ^2^*J*_CF_ = 31.7 Hz), 120.22, 119.02. FTIR (cm^−1^): 3101 (C_sp2_-H vinylic), 3027 (C_sp2_-H aromatic), 1654 (C=O), 1610 (C=C vinylic), 1527 (C=C aromatic), and 1108 (C-F). HRMS (ESI) *m*/*z*: 298.0511 [M + H]^+^ (calculated for C_14_H_10_F_3_NOS: 297.0435). HPLC purity = 99.9% (mobile phase: MeOH, 1 mL/min, tR = 2.351 min). m.p. 205–207 °C.

*(E)-3-(5-methylthiophen-2-yl)-N-phenylacrylamide* (**14**). The obtained compound was a yellow solid (105 mg, 43% yield). ^1^H NMR (400 MHz, DMSO-*d*_6_) δ: 10.13 (s, 1H), 7.69 (d, 2H), 7.65 (d, *J* = 13.1 Hz, 1H), 7.31 (t, *J* = 8.0, 8.0 Hz, 2H), 7.24 (d, *J* = 3.6 Hz, 1H), 7.05 (t, *J* = 7.4, 7.4 Hz, 1H), 6.83 (d, *J* = 2.4 Hz, 1H), 6.44 (d, *J* = 15.3 Hz, 1H), 2.47 (s, 3H). ^13^C NMR (101 MHz, DMSO-*d*_6_) δ: 163.42, 142.50, 139.35, 137.72, 133.51, 131.91, 128.83, 128.79, 126.95, 123.24, 119.53, 119.10, 119.01, 15.43. FTIR (cm^−1^): 3242 (N-H), 3189 (C_sp2_-H vinylic), 3045 (C_sp2_-H aromatic), 2924 (C_sp3_-H methyl), 1650 (C=O), 1606 (C=C vinylic), and 1541 (C=C aromatic) bands. HRMS (ESI) *m*/*z*: 244.0794 [M + H]^+^ (calculated for C_14_H_13_NOS: 243.0718). HPLC purity = 99.9% (mobile phase: MeOH, 1 mL/min, tR = 2.404 min). m.p. 175–177 °C.

*(E)-3-(5-methylthiophen-2-yl)-N-(4 (trifluoromethoxy)phenyl)acrylamide* (**15**). The obtained compound was a white solid (219 mg, 67% yield). ^1^H NMR (400 MHz, DMSO-*d*_6_) δ: 10.33 (s, 1H), 7.78 (d, *J* = 9.1 Hz, 2H), 7.68 (d, *J* = 15.3 Hz, 1H), 7.32 (d, *J* = 9.0 Hz, 2H), 7.26 (d, *J* = 3.6 Hz, 1H), 6.83 (d, *J* = 2.4 Hz, 1H), 6.41 (d, *J* = 15.3 Hz, 1H), 2.47 (s, 3H). ^13^C NMR (101 MHz, DMSO-*d*_6_) δ: 163.59, 143.52, 142.75, 138.57, 137.60, 133.97, 132.16, 127.00, 121.70, 121.43, 120.39, 120.16 (q ^1^*J*_CF_ = 256.4 Hz), 119.08, 118.89, 15.43. FTIR (cm^−1^): 3256 (N-H), 3250 (C_sp2_-H vinylic), 3059 (C_sp2_-H aromatic), 2932 (C_sp3_-H methyl), 1664 (C=O), 1615 (C=C vinylic), 1546 (C=C aromatic), 1264 (C-O), and 1165 (C-F) bands. HRMS (ESI) m/z: 328.0613 [M + H]^+^ (calculated for C_15_H_12_F_3_NO_2_S: 327.0541). HPLC purity = 99.9% (mobile phase: MeOH, 1 mL/min, tR = 2.397 min). m.p. 161–163 °C.

*(E)-3-(5-methylthiophen-2-yl)-N-(4-(trifluoromethyl)phenyl)acrylamide* (**16**). The obtained compound was a white solid (227 mg, 73% yield). ^1^H NMR (400 MHz, DMSO-*d*_6_) δ: 10.48 (s, 1H), 7.88 (d, *J* = 8.7 Hz, 2H), 7.76–7.70 (m, 1H), 7.68 (d, *J* = 6.8 Hz, 2H), 7.28 (d, *J* = 3.4 Hz, 1H), 6.84 (d, *J* = 3.5 Hz, 1H), 6.43 (d, *J* = 15.3 Hz, 1H), 2.48 (s, 3H). ^13^C NMR (101 MHz, DMSO-*d*_6_) δ: 163.94, 142.99, 137.53, 134.43, 132.43, 127.06, 126.12 (q ^3^*J*_CF_ = 3.70 Hz), 123.16 (q ^2^*J*_CF_ = 31.9 Hz), 119.08, 118.98, 118.86, 15.45. FTIR (cm^−1^): 3252 (N-H), 3126 (C_sp2_-H vinylic), 3045 (C_sp2_-H aromatic), 2934 (C_sp3_-H methyl), 1655 (C=O), 1606 (C=C vinylic), 1538 (C=C aromatic), and 1114 (C-F). HRMS (ESI) m/z: 312.0653 [M + H]^+^ (calcd C_15_H_12_F_3_NOS: 311.0592). HPLC purity = 99.9% (mobile phase: MeOH, 1 mL/min, tR = 2.405 min). m.p. 194–196 °C.

### 3.4. In Silico Prediction of Drug-like and Toxicological Properties

Prior to chemical synthesis, all designed *N*-phenyl-3-(thiophen-2-yl)acrylamide derivatives were subjected to in silico evaluation to assess their drug-like properties, physicochemical properties, and potential toxicological risks. This preliminary screening step was implemented to guide compound selection and ensure that the proposed molecules exhibited suitable characteristics for further evaluations.

The physicochemical parameters related to oral bioavailability were calculated using the SwissADME online platform [20]. Molecular descriptors, including the molecular weight (MW), iLogP, number of HBD, HBA, TPSA, and RB, were also determined. Drug-likeness was evaluated according to Lipinski’s rule of five, which is widely used to predict the oral bioavailability of small molecules [18].

Toxicological predictions were performed using the DataWarrior software (v06.05.01), which integrates fragment-based and empirical models to identify potential MUT, IRT, TUM, and REP risks [21]. The absence of predicted toxicological alerts was considered an essential criterion for prioritizing the compounds for synthesis.

The calculated parameters are presented in Table 1. Only compounds that fulfilled Lipinski’s criteria and showed favorable toxicity profiles were selected for chemical synthesis. This in silico filtering strategy was employed to reduce the likelihood of poor pharmacokinetic behavior or early toxicity, thereby strengthening the link between rational molecular design, experimental synthesis, and biological testing.

### 3.5. Cell Culture

Cell lines. The established human tumor cell lines AGS (human gastric adenocarcinoma cells; Cat. No. 89090402) and MCF-7 (Cat. No. 86012803) were obtained from the European Collection of Authenticated Cell Cultures (ECACC, Salisbury, UK). CACO-2 (Cat. No. HTB-37), HepG2 (catalog number: HB-8065), and HDF (Cat. No. PCS-201-012) were obtained from American Type Culture Collection (ATCC, Manassas, VA, USA). HSC-3 (human tongue squamous cell carcinoma cells; Cat. No. SCC193) was obtained from Merck. HDFa cells were used as the non-tumorigenic control. All cell lines were used according to the suppliers’ recommendations. These cell lines differ in terms of tissue origin, biological characteristics, and drug response profiles, allowing the evaluation of the synthesized compounds across a diverse range of cancer types and providing a broader assessment of their antiproliferative potential.

All cell lines were cultured and expanded in Dulbecco’s Modified Eagle’s Medium (DMEM, Gibco, Thermo Fisher Scientific, Waltham, MA, USA) supplemented with 10% Fetal Bovine Serum (Biological Industries, Beit Haemek, Israel) and 1% penicillin–streptomycin antibiotic mixture (Biological Industries) at 37 °C in a 5% CO_2_ and 95% air atmosphere.

### 3.6. Cytotoxicity Assays

The cytotoxic effects of the compounds were evaluated using the SRB assay [26]. Cells were seeded in 96-well microplates at a density of 2 × 10^4^ cells/well. All compounds were initially dissolved in 100% DMSO, prediluted to reduce the DMSO concentration to 1% or lower in the culture medium, and added to the cells at different concentrations for 24 h. Cells were fixed by adding 10% (*w*/*v*) trichloroacetic acid (TCA) (Winkler, Heidelberg, Germany) to each well and stained with 0.057% (*w*/*v*) SRB (Sigma-Aldrich) in 1% (*v*/*v*) acetic acid (Winkler) solution. The bound dye was dissolved in 10 mM Tris base (Sigma-Aldrich), and the absorbance was measured at 510 nm using a multiplate reader (Synergy 2, Biotek Instruments). Percentage cell viability was determined using the following formula: cell viability (%) = (absorbance of treated cells/absorbance of untreated cells) × 100. The absorbance of the wells of the negative control (1% DMSO in culture medium) was considered to represent 100% viability.

## 4. Conclusions

In this study, a series of *N*-phenyl-3-(thiophen-2-yl)acrylamide derivatives (**11**–**16**) were synthesized and evaluated for cytotoxic activity against a panel of human cancer cell lines. The objective of this study was to investigate the influence of structural modifications on the phenyl and thiophene rings on the biological properties of this scaffold. Among the evaluated compounds, derivatives **11** and **16** exhibited the most consistent antiproliferative activity while maintaining low toxicity toward non-cancerous HDF. Although the observed cytotoxicity was relatively weak, the results revealed preliminary SAR analysis, suggesting that the combination of a para-CF_3_ substituent and a 5-methylthiophene moiety may be beneficial within this limited compound series. Overall, these findings provide an initial assessment of the *N*-phenyl-3-(thiophen-2-yl)acrylamide scaffold and support further structural optimization to improve potency and better define its anticancer potential.

## Figures and Tables

**Table 2:** Data are presented as mean ± SD from at least three independent experiments performed in triplicate. ND = not determined (compounds that did not reach 50% inhibition were not further evaluated in HDF cells). Values for compounds **12**–**15** are included for completeness; however, reliable IC_50_ determination was only possible under the specific conditions indicated.

Compound	AGS (µM)	Caco-2 (µM)	MCF-7 (µM)	HDF (Non-Cancerous) (µM)
**11**	379 ± 18	420 ± 22	265 ± 14	>1000
**16**	272 ± 15	256 ± 12	242 ± 11	>1000
**12**	>1000	>1000	954 ± 45	ND
**13**	>1000	>1000	474 ± 28	ND
**14**	906 ± 52	>1000	>1000	ND
**15**	>1000	>1000	>1000	ND
**Doxorubicin**	0.45 ± 0.03	0.52 ± 0.04	0.38 ± 0.02	2.8 ± 0.2

## Data Availability

The original contributions presented in this study are included in the article/Supplementary Materials. Further inquiries can be directed to the corresponding author(s).

## Supplementary Materials

The following supporting information can be downloaded at: https://www.mdpi.com/article/10.3390/molecules31183147/s1. PDF file containing FTIR Spectra of Compounds; NMR Spectra of Compounds; Absorption Spectra of Compounds; HRMS (QqTOF) Spectra of Compounds.
