# Supplementary material for: Design, Synthesis, and Cytotoxic Evaluation of N-Phenyl-3-(Thiophen-2-Yl)acrylamide Derivatives
Source: Molecules. 2026 Sep 8;31(18):3147. doi: 10.3390/molecules31183147 (PMC13609584; doi:10.3390/molecules31183147)
Supplement: Supplementary file 1 [file molecules-31-03147-s001.zip › molecules-4523249-supplementary.pdf]

Design, Synthesis, and Cytotoxic Evaluation of *N*-Phenyl-3-(Thiophen-2-yl)acrylamide Derivatives

Pablo S. Cavagnero,[1] Felipe A. Zúñiga,[2] Yaritza Ormazábal,[2] Yeray A. Rodríguez-Núñez,[3] David Ramírez,[4] Paulina V. Hormazabal,[4] Jorge Saavedra-Olavarría,[5] Claudio A. Jiménez,[1*] Efraín Polo-Cuadrado[1*] and Jhon J. López[5*]

[1]Universidad de Concepción, Facultad de Ciencias Químicas, Departamento de Química Orgánica, Concepción 4130000, Chile.

[2]Universidad de Concepcion, Facultad de Farmacia, Departamento de Bioquímica Clínica e Inmunología, Concepcion 4130000, Chile.

[3]Universidad Andrés Bello, Facultad de Ciencias Exactas, Departamento de Ciencias Químicas, Santiago 8370146, Chile.
[4]Universidad de Concepción, Facultad de Ciencias Biológicas, Departamento de Farmacología, 4130000, Chile.

[5]Pontificia Universidad Católica de Chile, Facultad de Química y de Farmacia, Escuela de Química, Departamento de Química Orgánica, Santiago 7820436, Chile.

***Corresponding Author**

*Claudio A. Jiménez – ORCID: 0000-0002-2622-8308. E-mail: cjimenez@udec.cl
*Efraín A. Polo-Cuadrado – ORCID: 0000-0003-2280-9321. E-mail: epolo@udec.cl
*Jhon J. López – ORCID: 0000-0002-2678-3627. E-mail: jjlopez@uc.cl

Table of contents

FTIR SPECTRA OF COMPOUNDS........ S2
Figure S1. FTIR spectra of compound **6**........ S2
Figure S2. FTIR spectra of compound **7**........ S2
Figure S3. FTIR spectra of compound **11**........ S3
Figure S4. FTIR spectra of compound **12**........ S3
Figure S5. FTIR spectra of compound **13**........ S4
Figure S6. FTIR spectra of compound **14**........ S4
Figure S7. FTIR spectra of compound **15**........ S5
Figure S8. FTIR spectra of compound **16**........ S5
NMR SPECTRA OF COMPOUNDS........ S6
Figure S9. ^{1}H NMR spectra of compound **6** (400 MHz, $CDCl_3$)........ S6
Figure S10. ^{13}C NMR spectra of compound **6** (101 MHz, $CDCl_3$)........ S6
Figure S11. ^{1}H NMR spectra of compound **7** (400 MHz, $CDCl_3$)........ S7
Figure S12. ^{13}C NMR spectra of compound **7** (101 MHz, $CDCl_3$)........ S7
Figure S13. ^{1}H NMR spectra of compound **11** (400 MHz, DMSO-d_6)........ S8
Figure S14. ^{13}C NMR spectra of compound **11** (101 MHz, DMSO-d_6)........ S8
Figure S15. ^{1}H NMR spectra of compound **12** (400 MHz, DMSO-d_6)........ S9
Figure S16. ^{13}C NMR spectra of compound **12** (101 MHz, DMSO-d_6)........ S9
Figure S17. ^{1}H NMR spectra of compound **13** (400 MHz, DMSO-d_6)........ S10
Figure S18. ^{13}C NMR spectra of compound **13** (101 MHz, DMSO-d_6)........ S10
Figure S19. ^{1}H NMR spectra of compound **14** (400 MHz, DMSO-d_6)........ S11
Figure S20. ^{13}C NMR spectra of compound **14** (101 MHz, DMSO-d_6)........ S11
Figure S21. ^{1}H NMR spectra of compound **15** (400 MHz, DMSO-d_6)........ S12
Figure S22. ^{13}C NMR spectra of compound **15** (101 MHz, DMSO-d_6)........ S12
Figure S23. ^{1}H NMR spectra of compound **16** (400 MHz, DMSO-d_6)........ S13
Figure S24. ^{13}C NMR spectra of compound **16** (101 MHz, DMSO-d_6)........ S13
Figure S25. Absorption spectra of compounds **11-16**........ S14
Figure S26. Chromatograms of the separation of compounds **11**-**16** by HPLC........ S15
HRMS (QqTOF) SPECTRA OF COMPOUNDS........ S16
Figure S27. HRMS (QqTOF) m/z spectra **11**........ S16
Figure S28. HRMS (QqTOF) m/z spectra **12**........ S16
Figure S29. HRMS (QqTOF) m/z spectra **13**........ S17
Figure S30. HRMS (QqTOF) m/z spectra **14**........ S17
Figure S31. HRMS (QqTOF) m/z spectra **15**........ S18
Figure S32. HRMS (QqTOF) m/z spectra **16**........ S18

FTIR SPECTRA OF COMPOUNDS

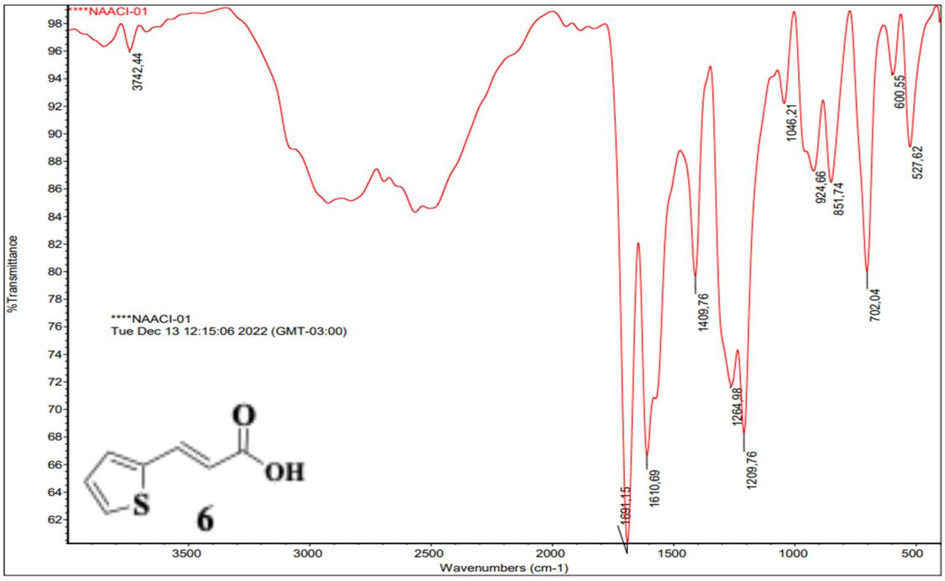

Figure S1. FTIR spectra of compound **6**.

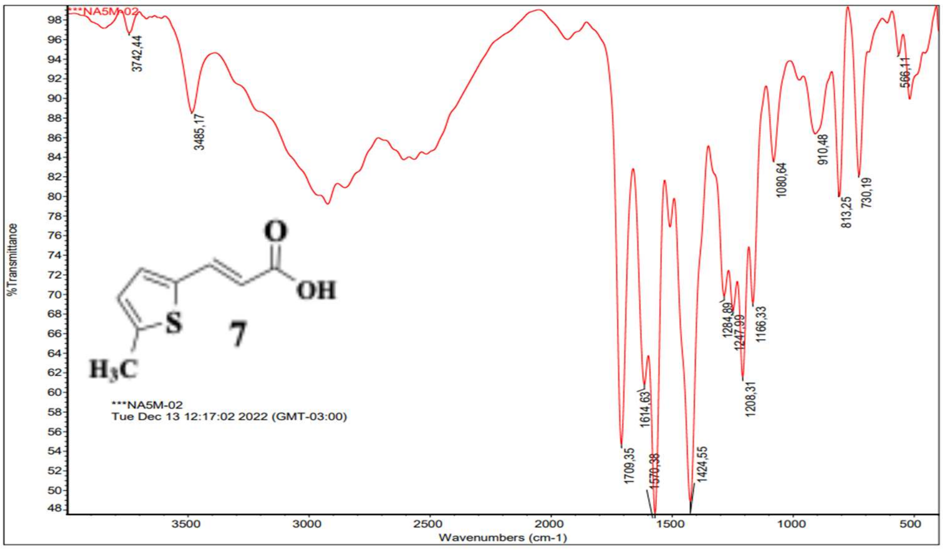

Figure S2. FTIR spectra of compound **7**.

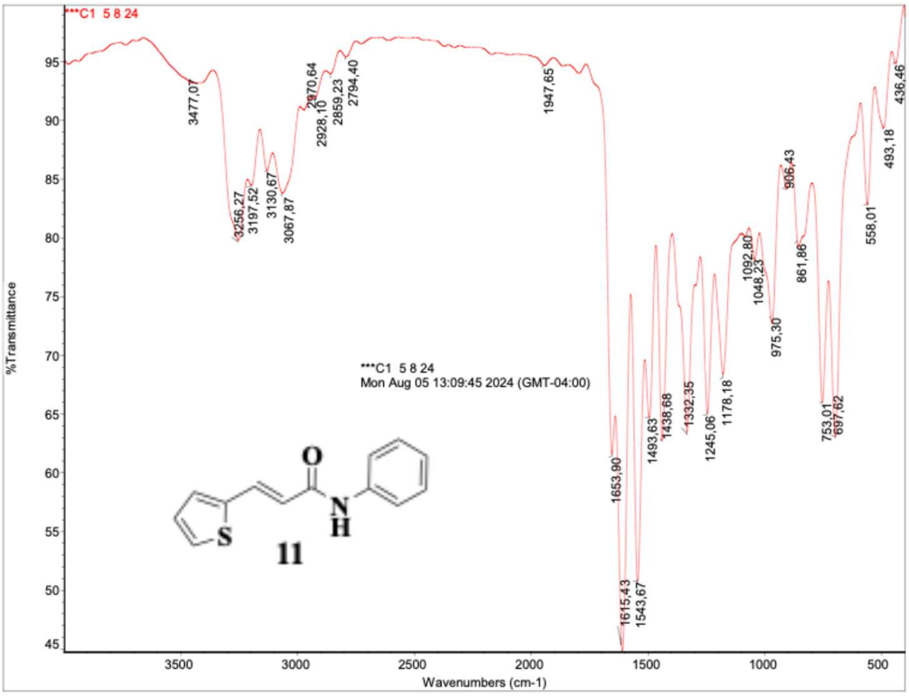

Figure S3. FTIR spectra of compound **11**.

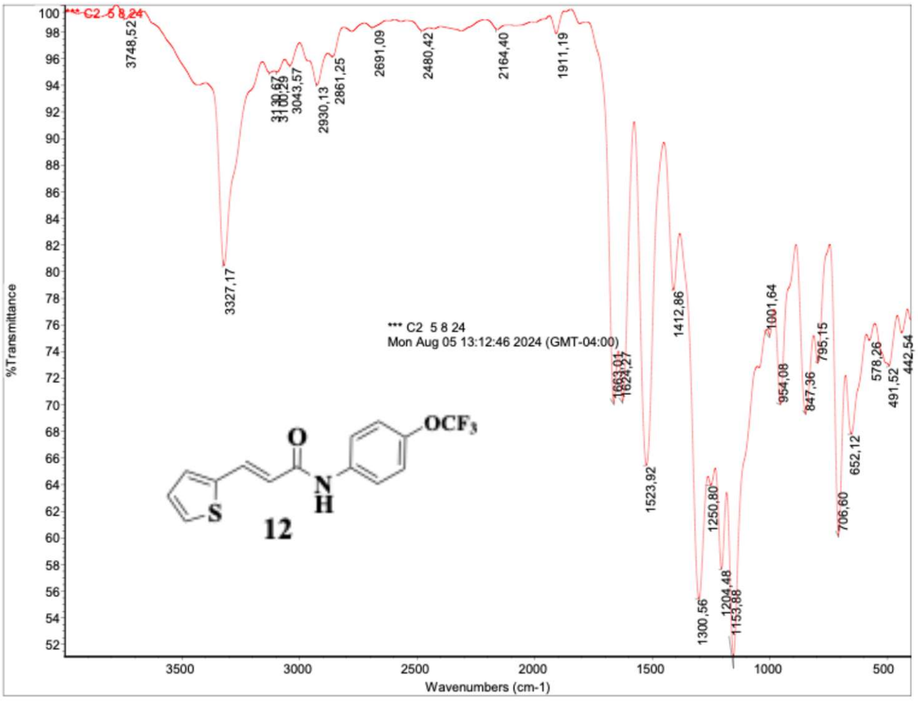

Figure S4. FTIR spectra of compound **12**.

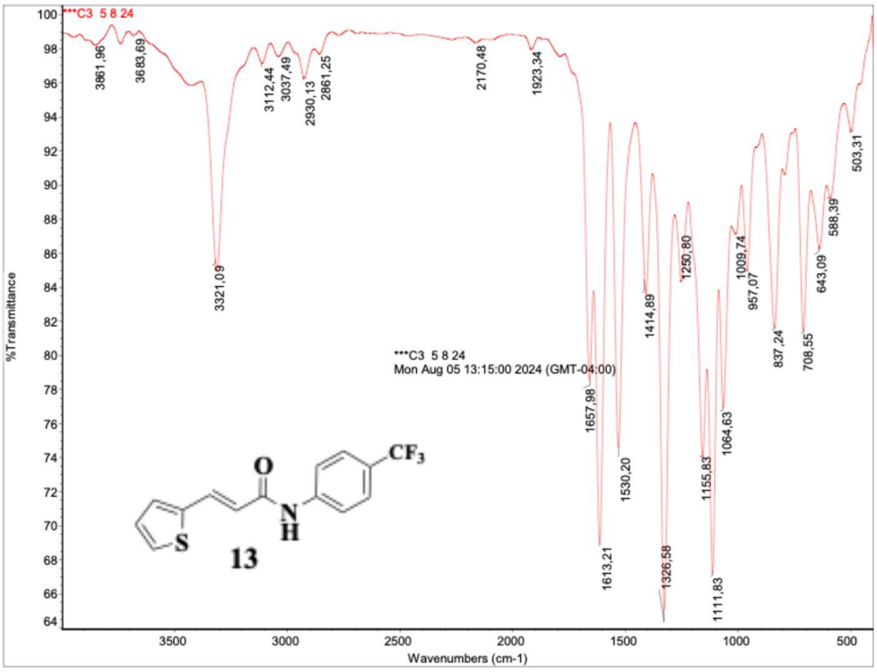

Figure S5. FTIR spectra of compound **13**.

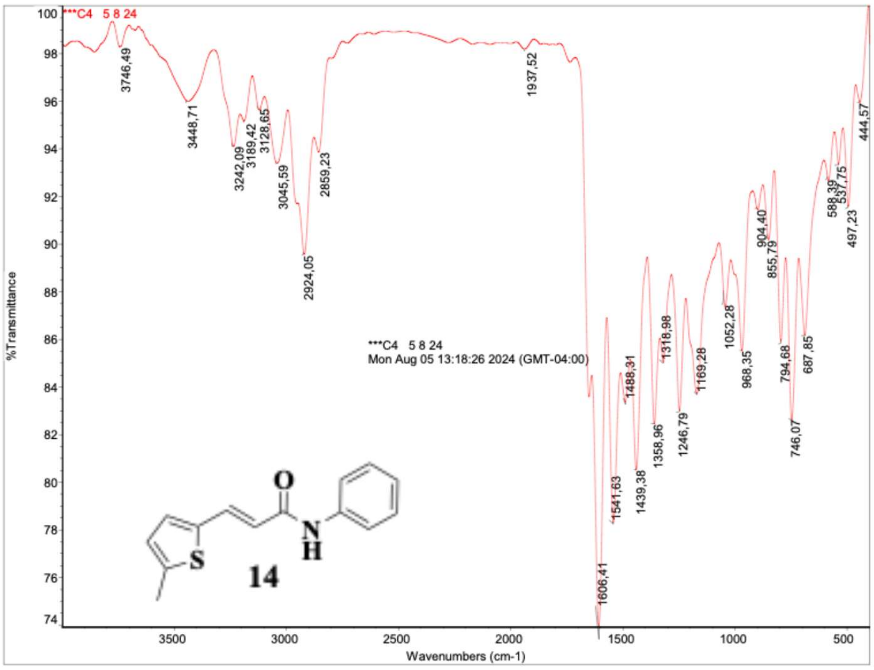

Figure S6. FTIR spectra of compound **14**.

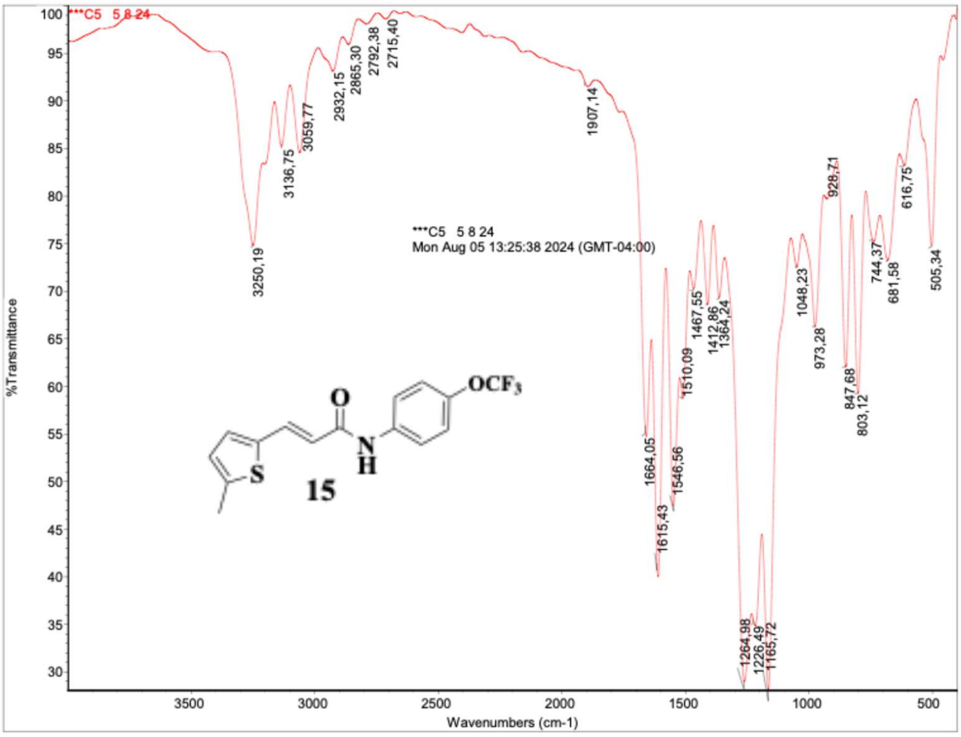

Figure S7. FTIR spectra of compound **15**.

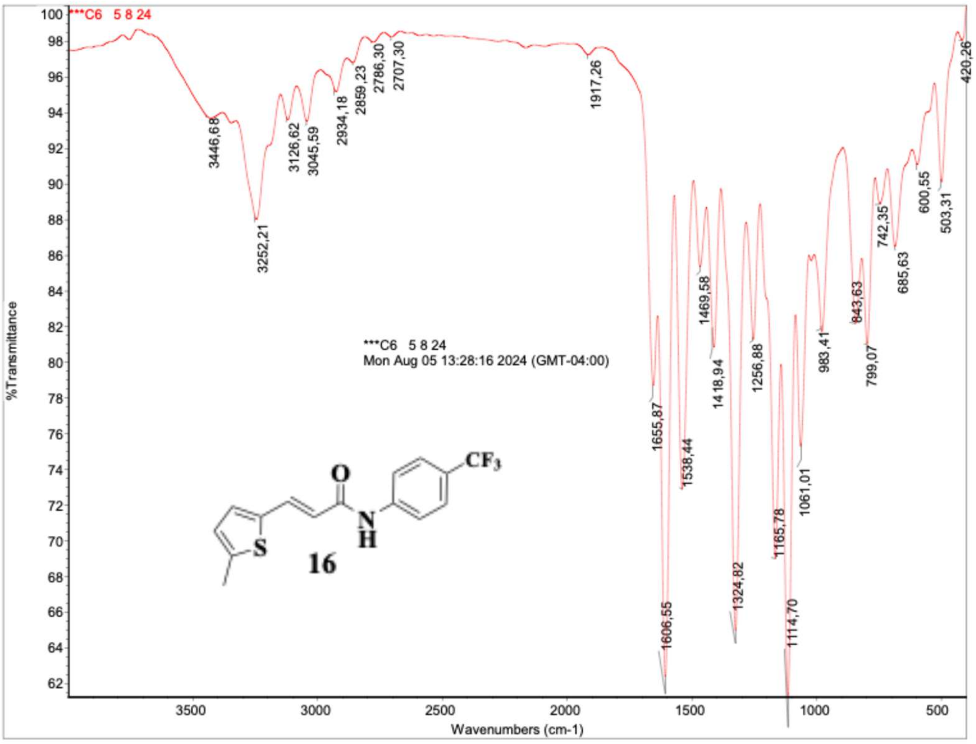

Figure S8. FTIR spectra of compound **16**.

NMR SPECTRA OF COMPOUNDS

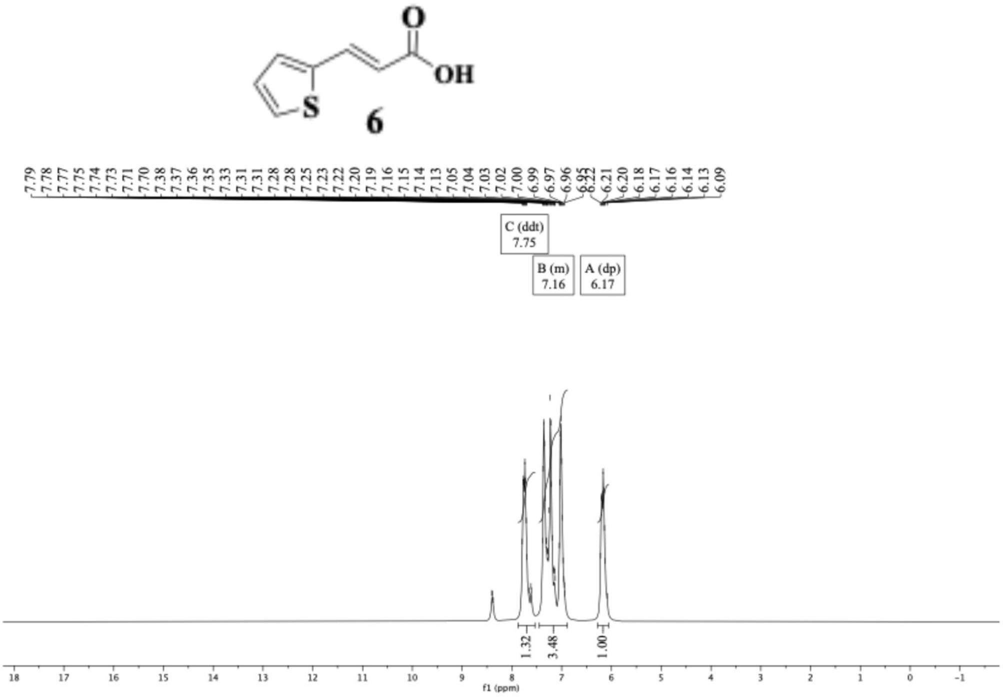

Figure S9. ^{1}H NMR spectra of compound **6** (400 MHz, $CDCl_3$).

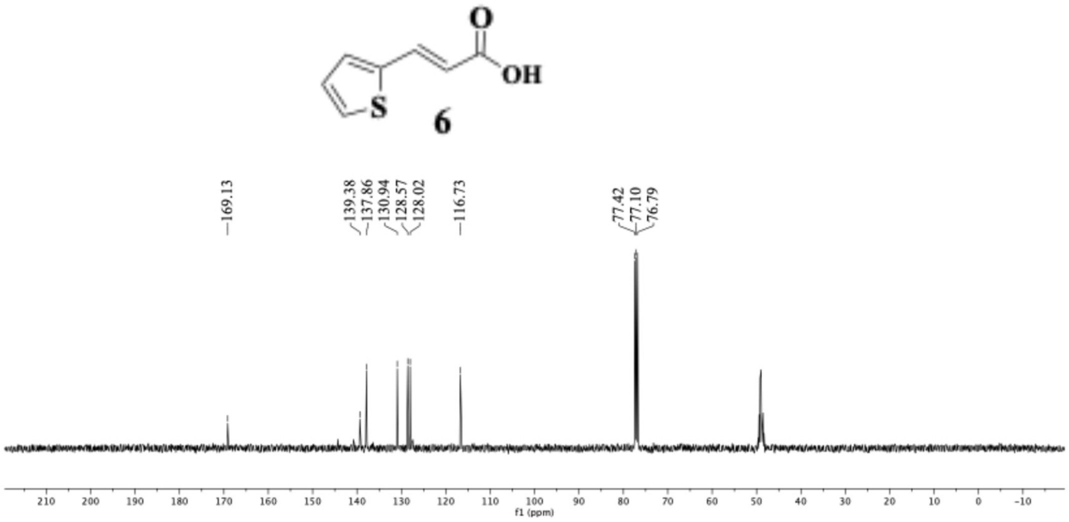

Figure S10. ^{13}C NMR spectra of compound **6** (101 MHz, $CDCl_3$).

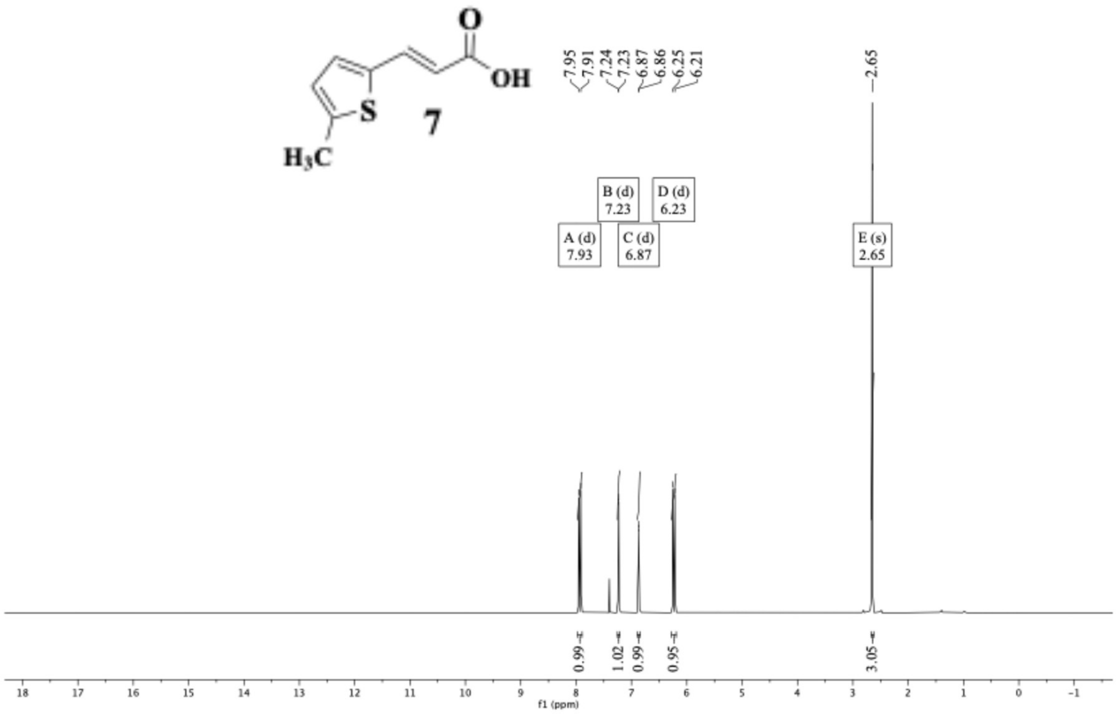

Figure S11. ^{1}H NMR spectra of compound **7** (400 MHz, $CDCl_3$).

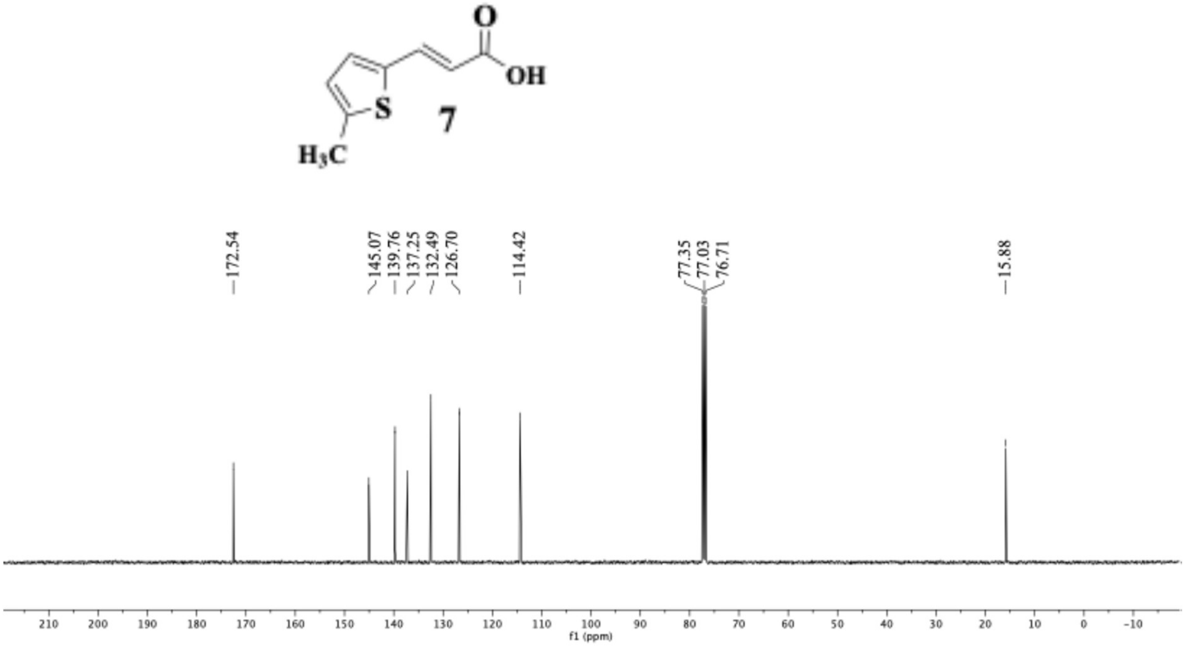

Figure S12. ^{13}C NMR spectra of compound **7** (101 MHz, $CDCl_3$).

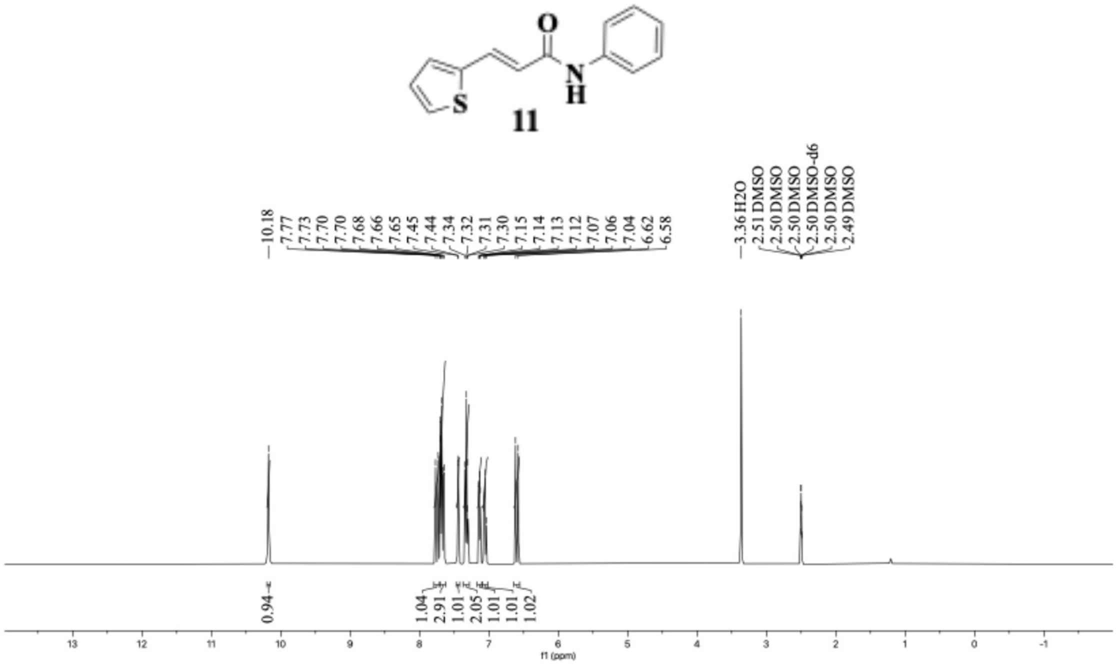

Figure S13. ^{1}H NMR spectra of compound **11** (400 MHz, DMSO-d_6).

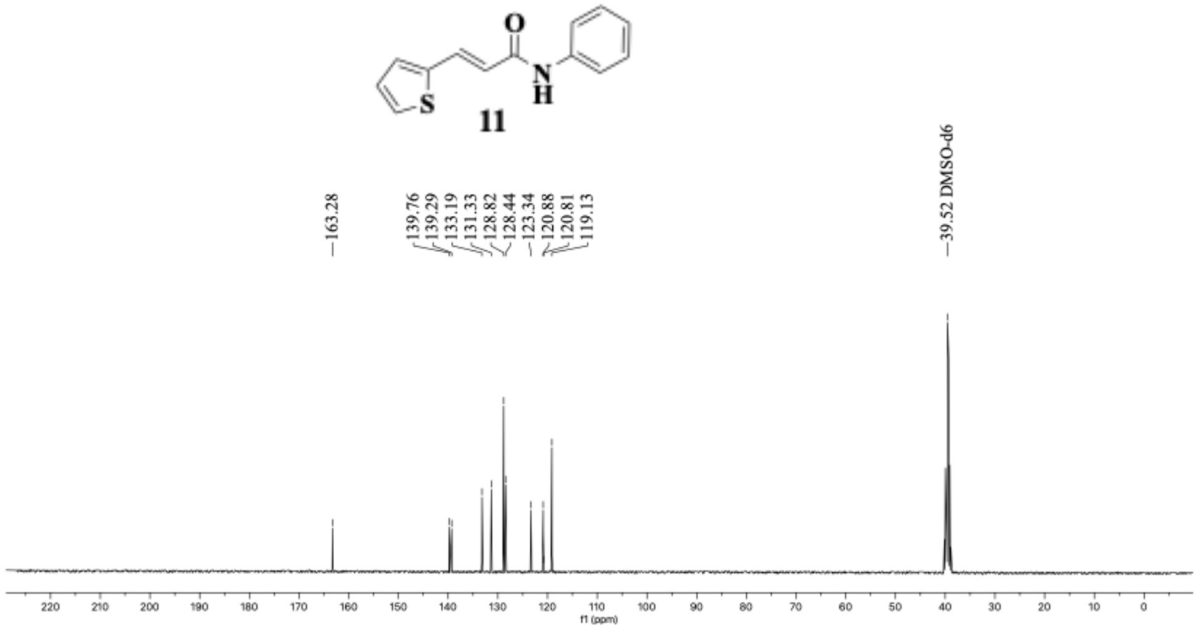

Figure S14. ^{13}C NMR spectra of compound **11** (101 MHz, DMSO-d_6).

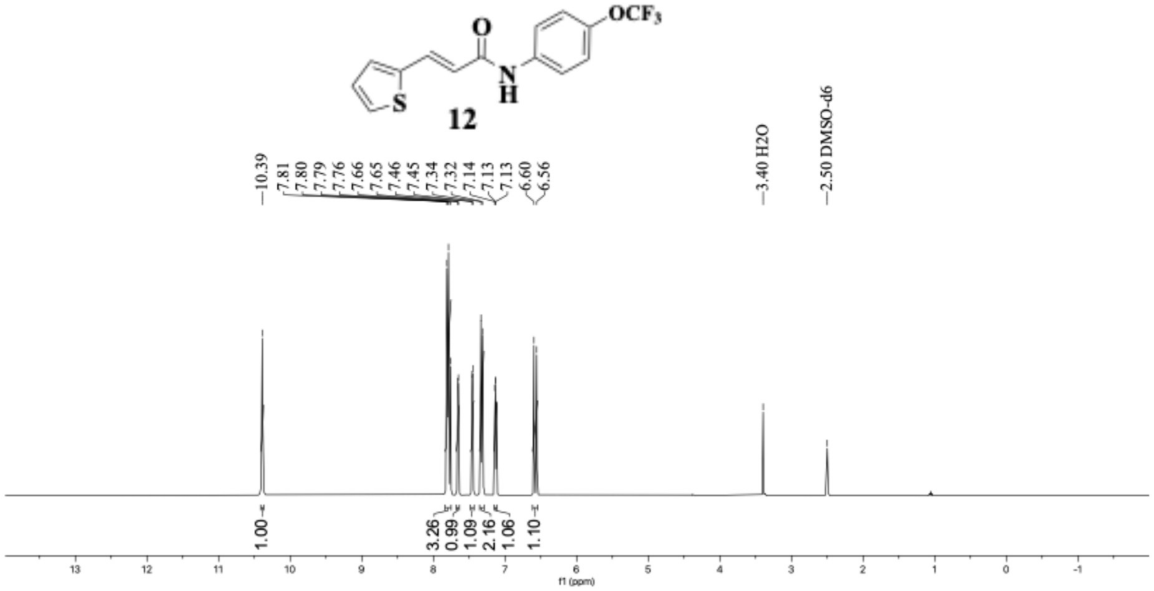

Figure S15. ^{1}H NMR spectra of compound **12** (400 MHz, DMSO-d_6).

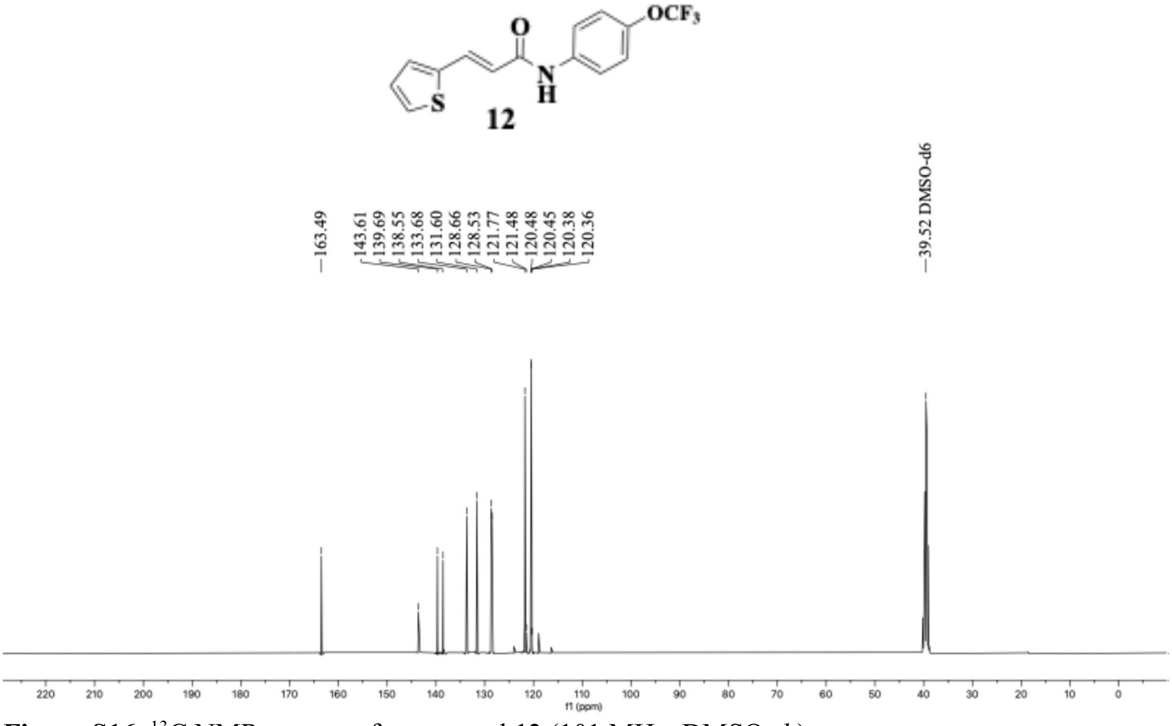

Figure S16. ^{13}C NMR spectra of compound **12** (101 MHz, DMSO-d_6).

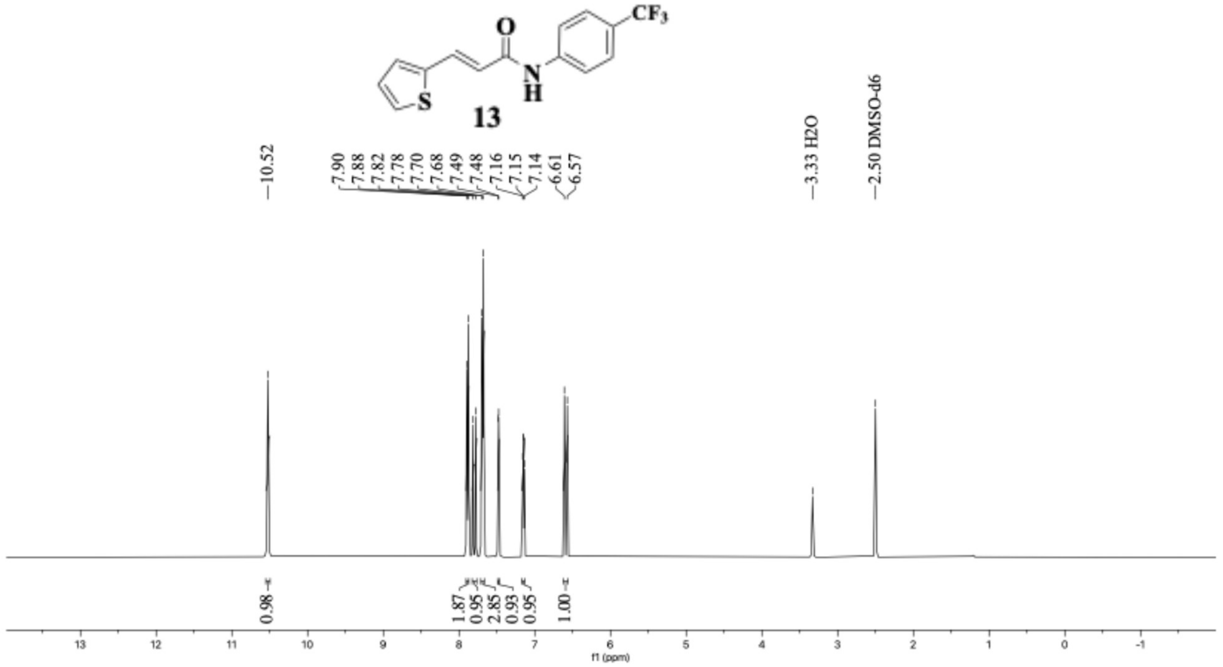

Figure S17. ^{1}H NMR spectra of compound **13** (400 MHz, DMSO-d_6).

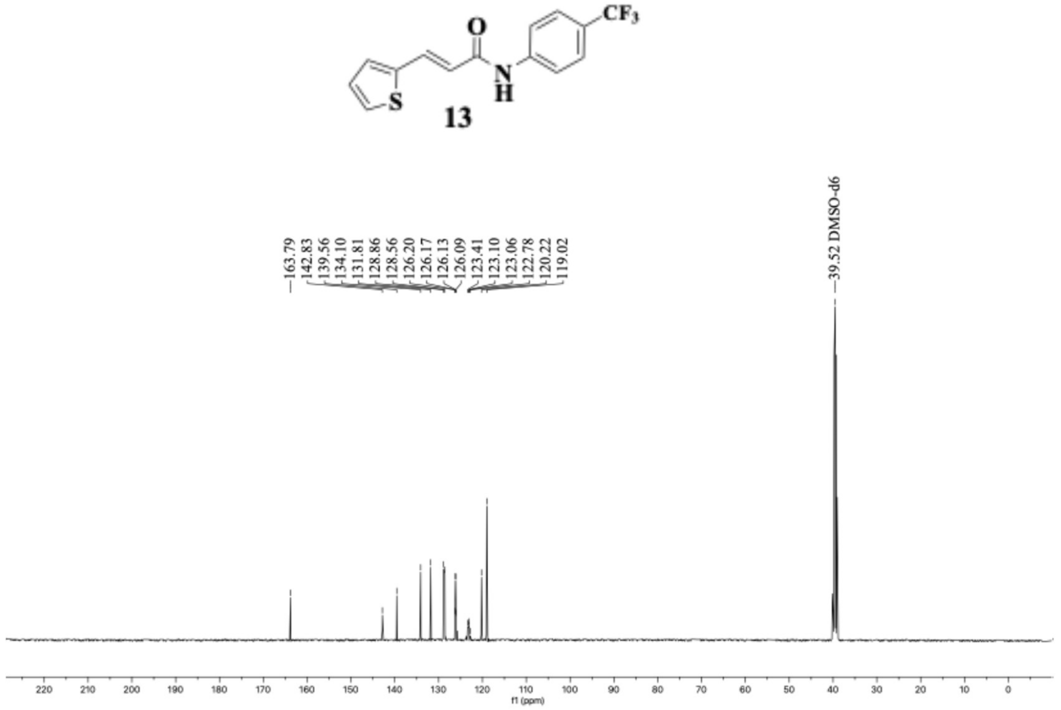

Figure S18. ^{13}C NMR spectra of compound **13** (101 MHz, DMSO-d_6).

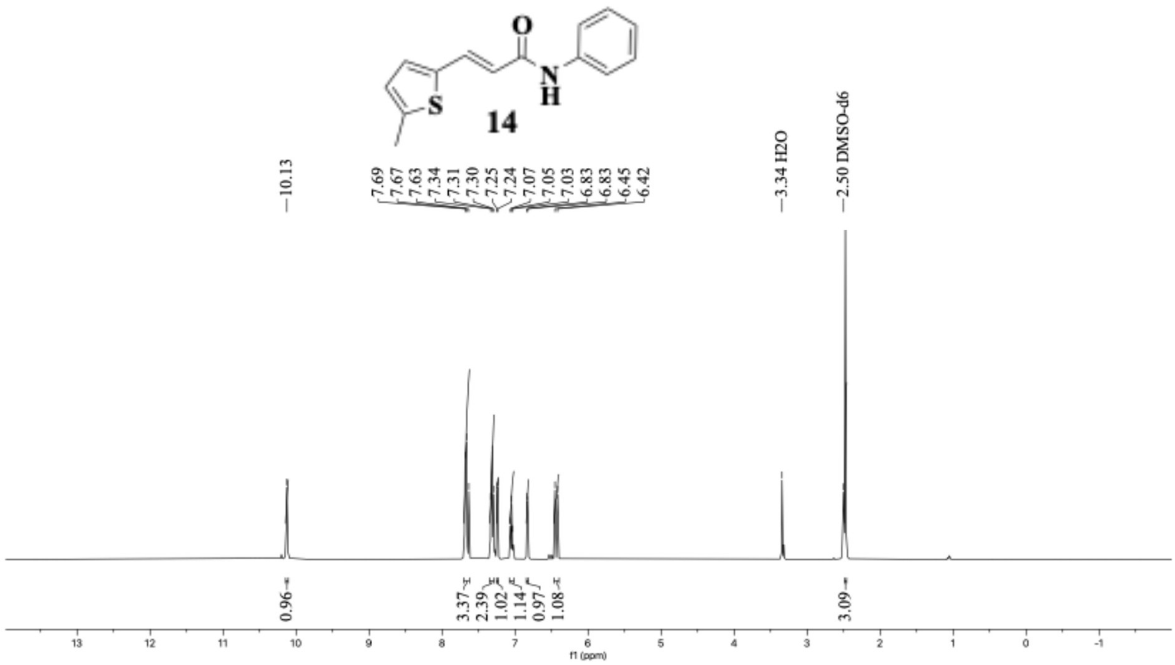

Figure S19. ^{1}H NMR spectra of compound **14** (400 MHz, DMSO-d_6).

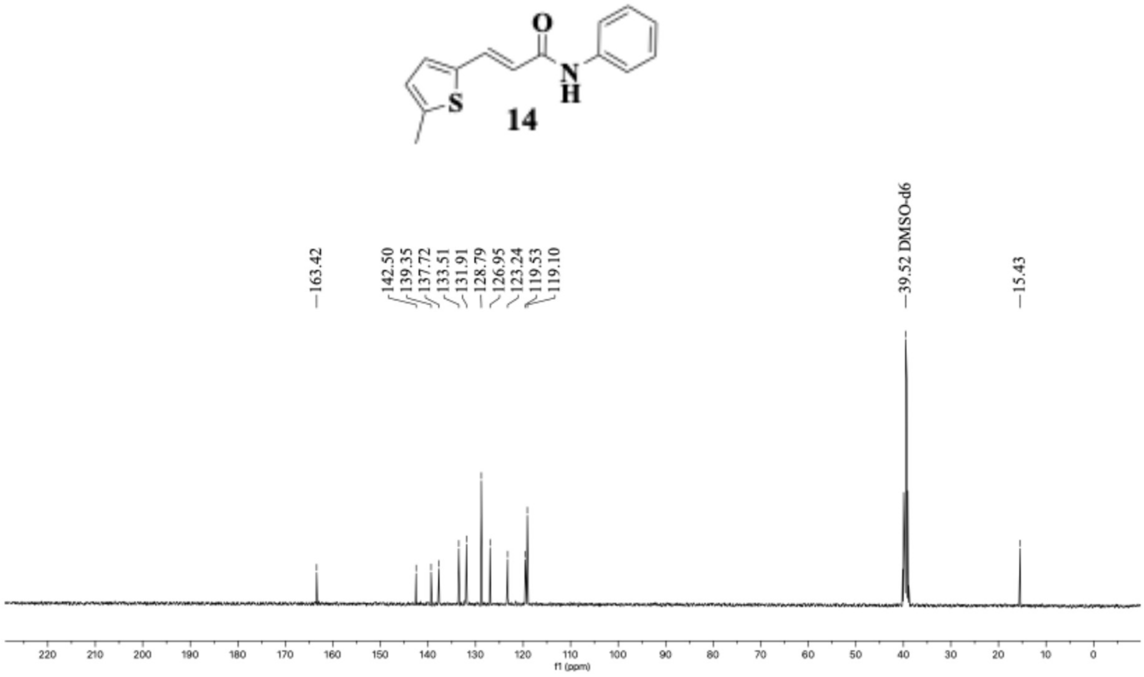

Figure S20. ^{13}C NMR spectra of compound **14** (101 MHz, DMSO-d_6).

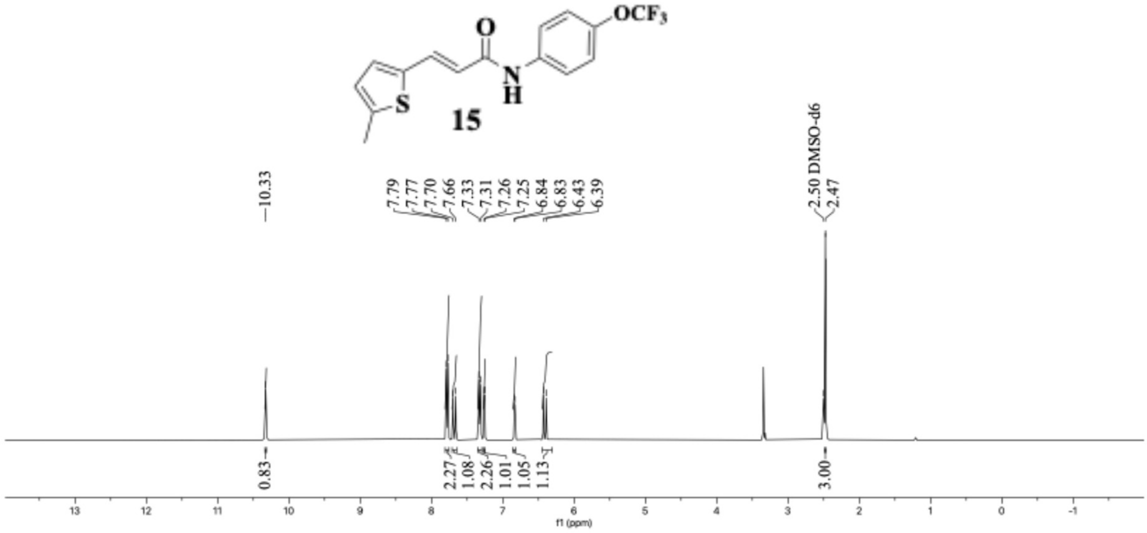

Figure S21. ^{1}H NMR spectra of compound **15** (400 MHz, DMSO-d_6).

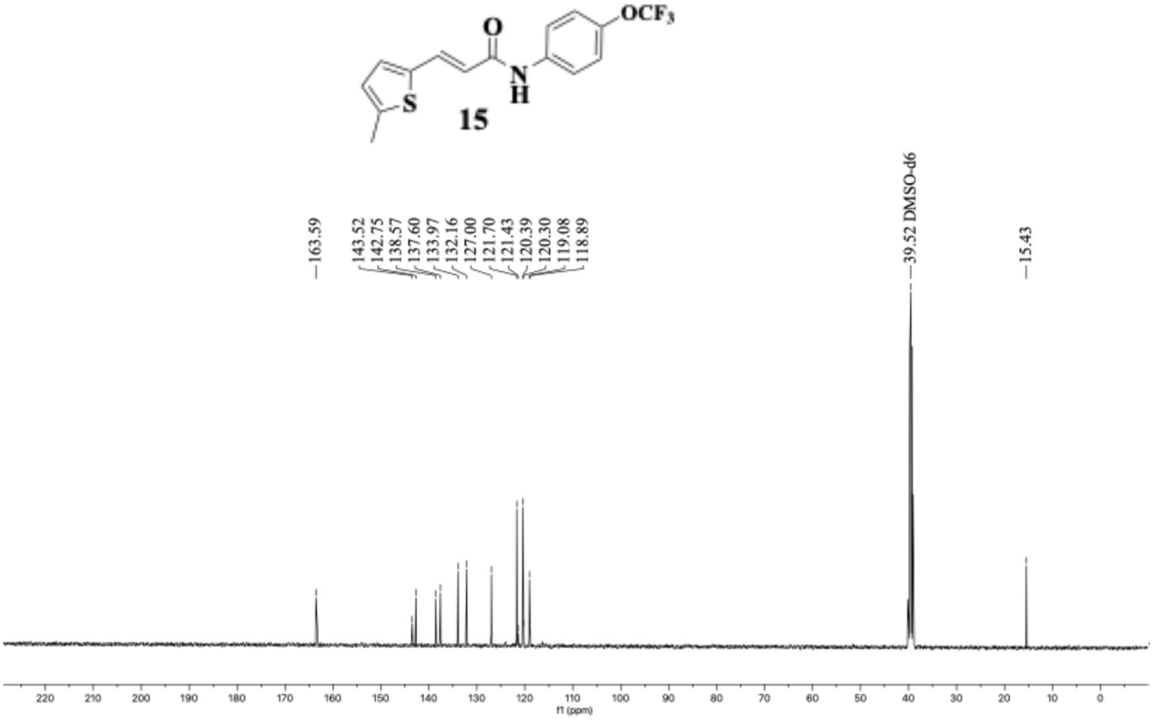

Figure S22. ^{13}C NMR spectra of compound **15** (101 MHz, DMSO-d_6).

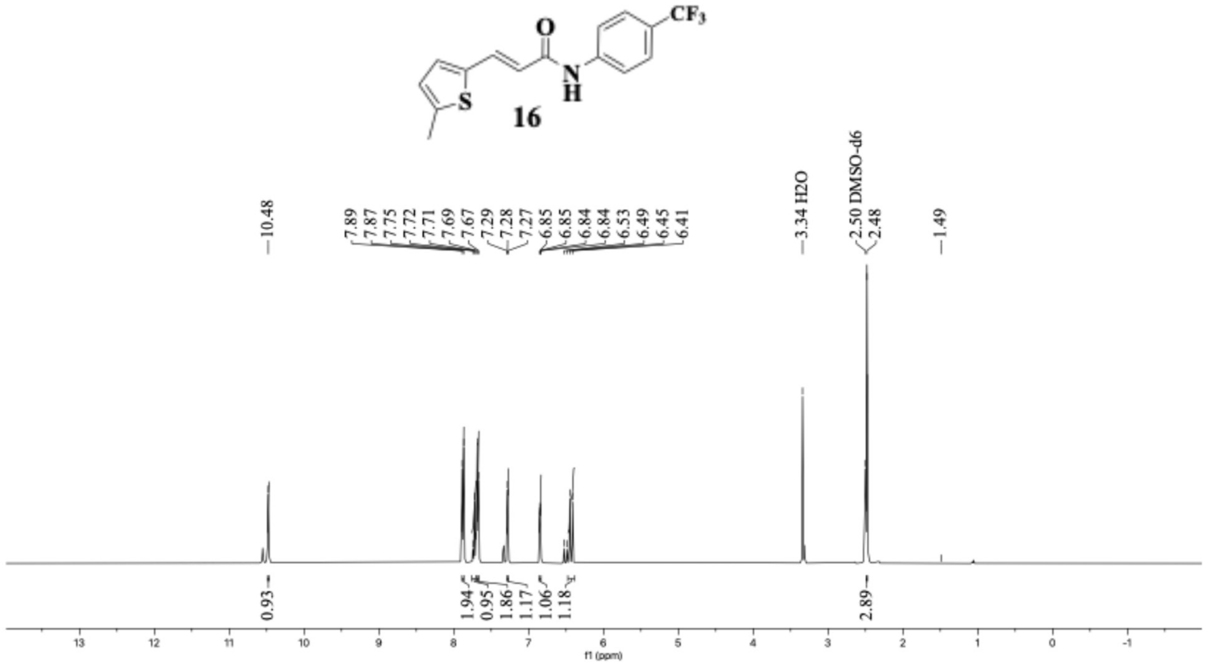

Figure S23. ^{1}H NMR spectra of compound **16** (400 MHz, DMSO-d_6).

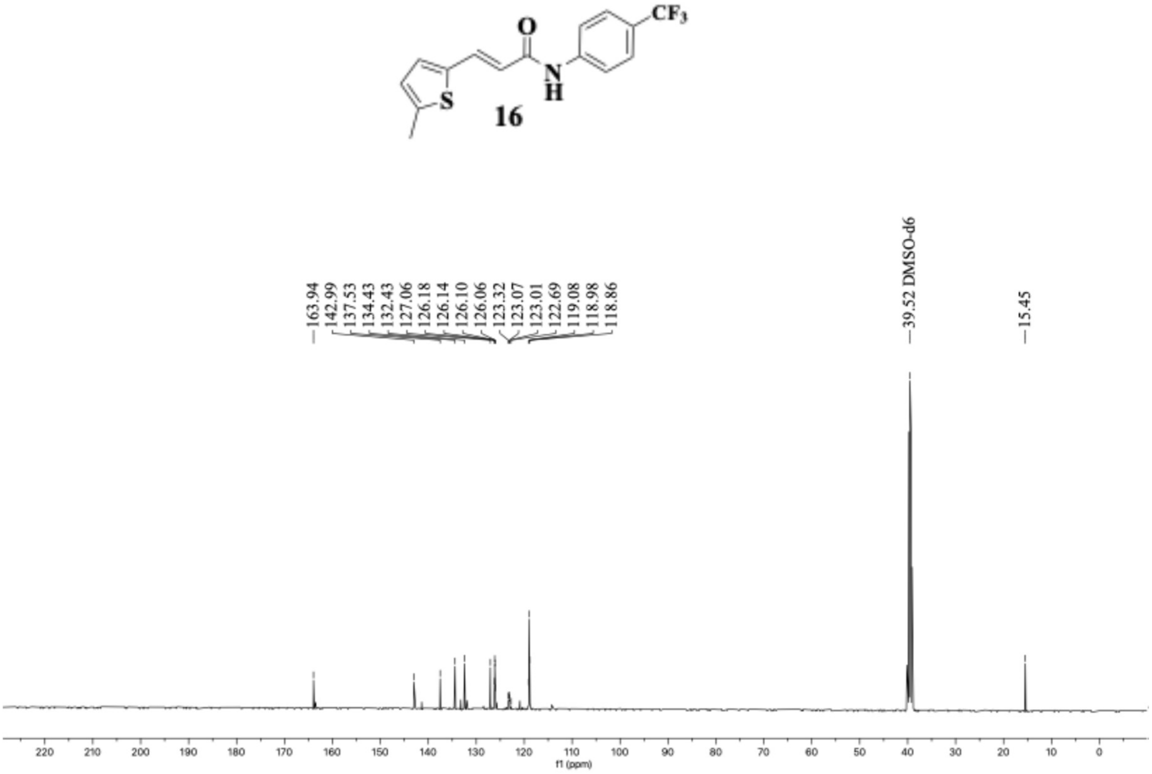

Figure S24. ^{13}C NMR spectra of compound **16** (101 MHz, DMSO-d_6).

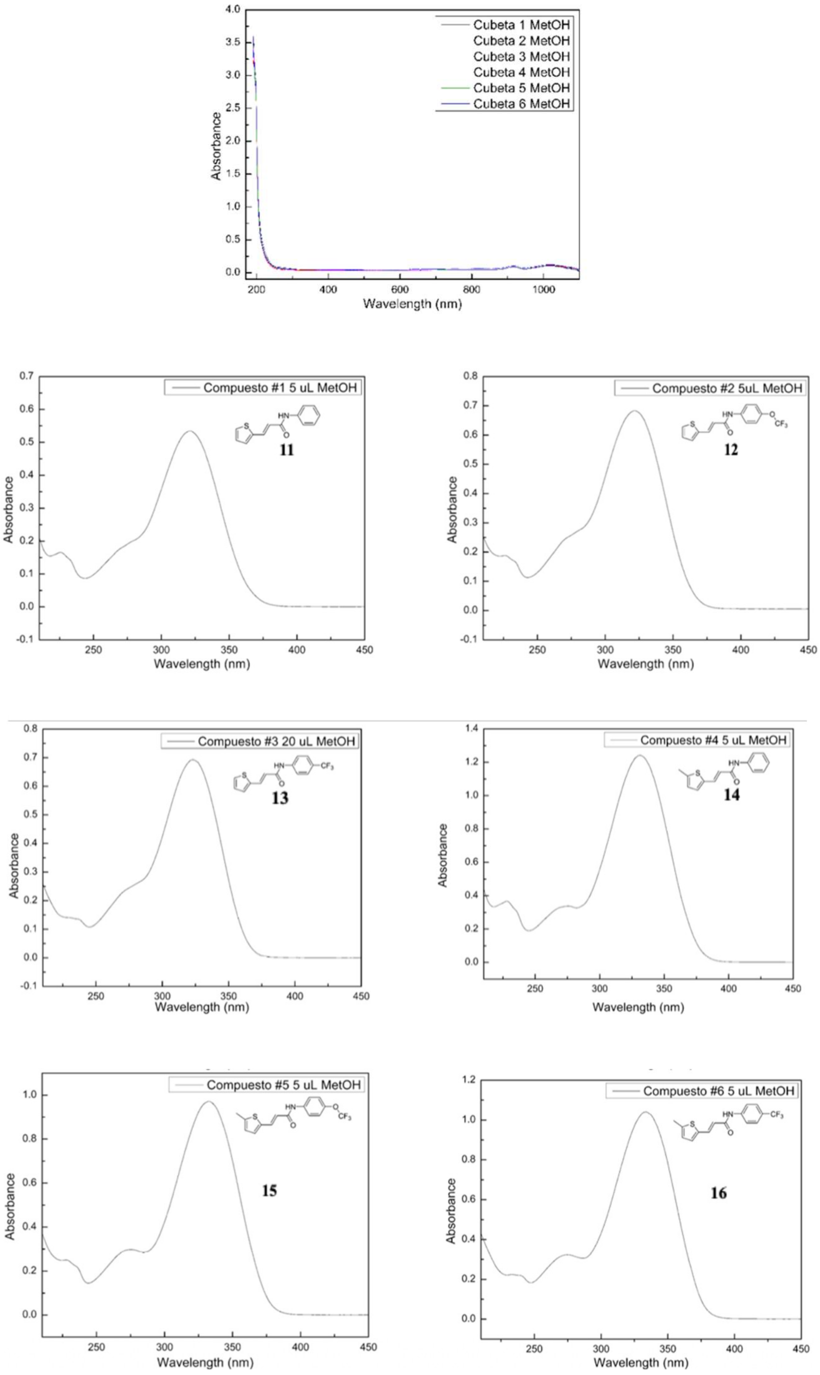

Figure S25. Absorption spectra of compounds **11-16**.

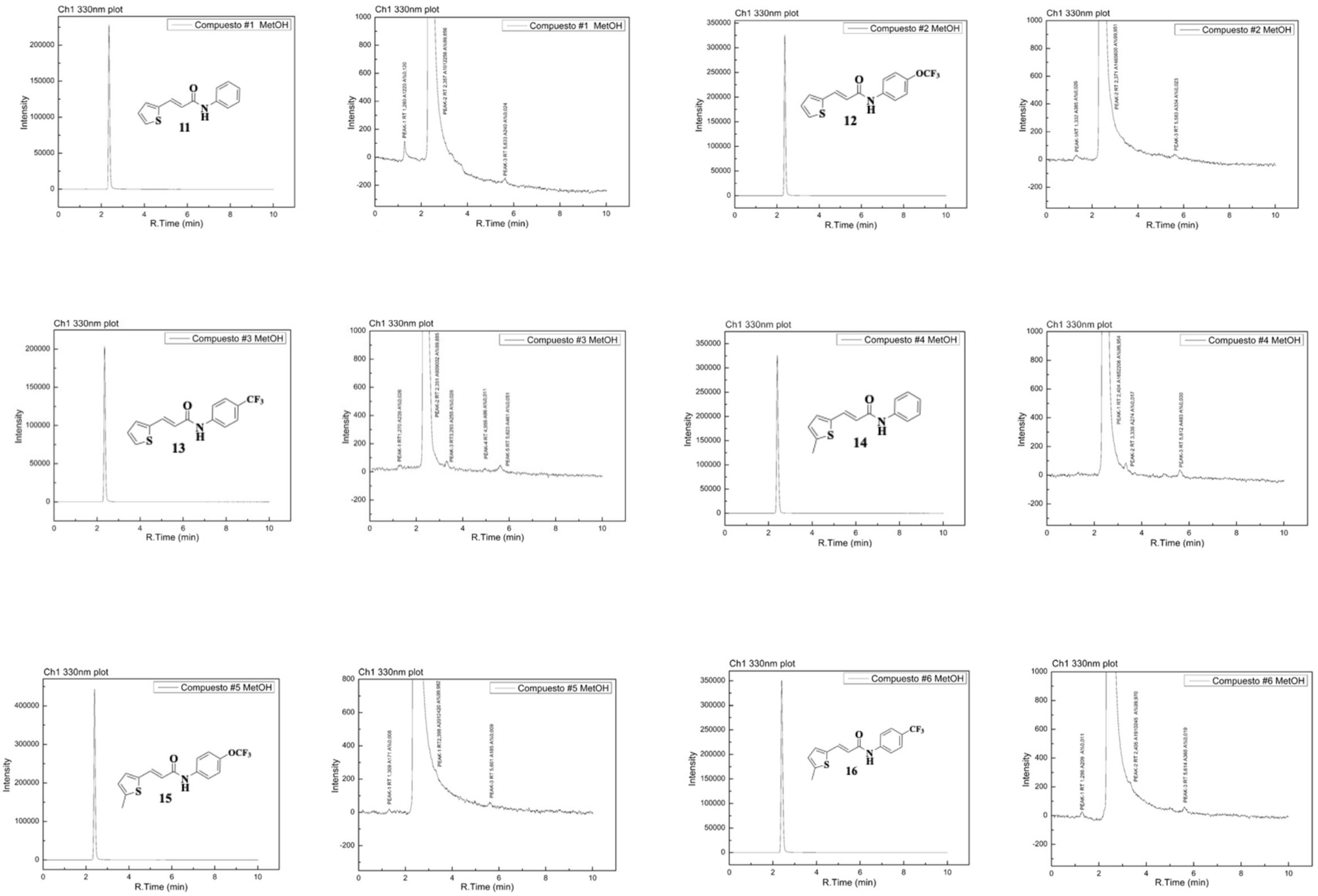

Figure S26. Chromatograms of the separation of compounds **11-16** by HPLC.

HRMS (QqTOF) SPECTRA OF COMPOUNDS

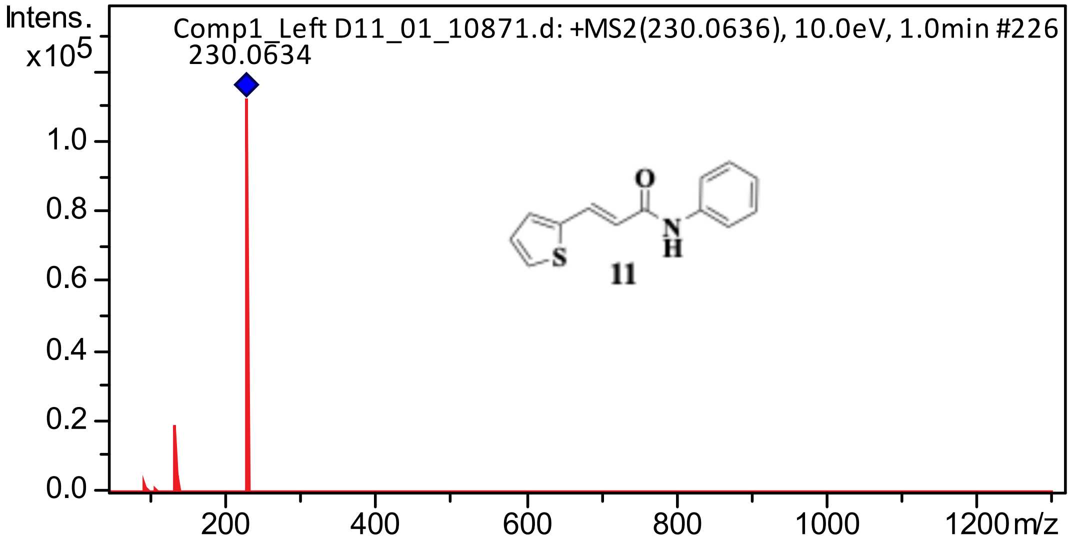

Figure S27. HRMS (QqTOF) m/z spectra **11**.

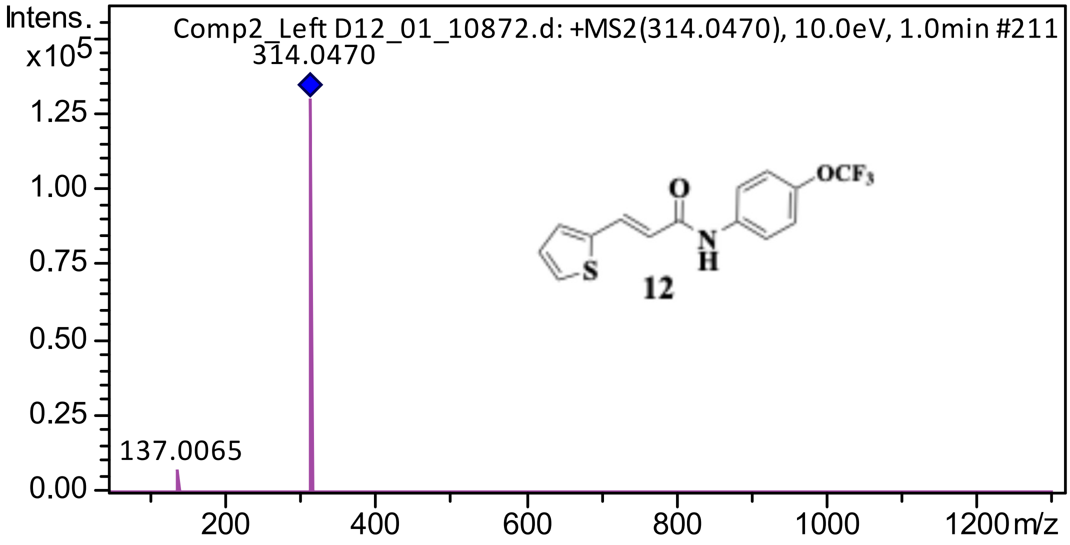

Figure S28. HRMS (QqTOF) m/z spectra **12**.

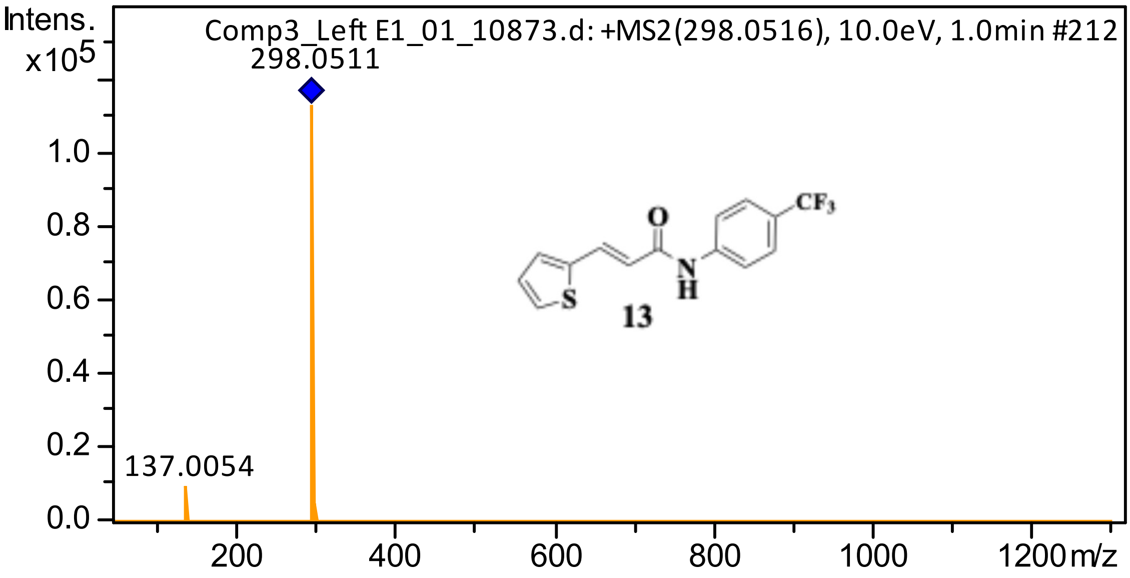

Figure S29. HRMS (QqTOF) m/z spectra **13**.

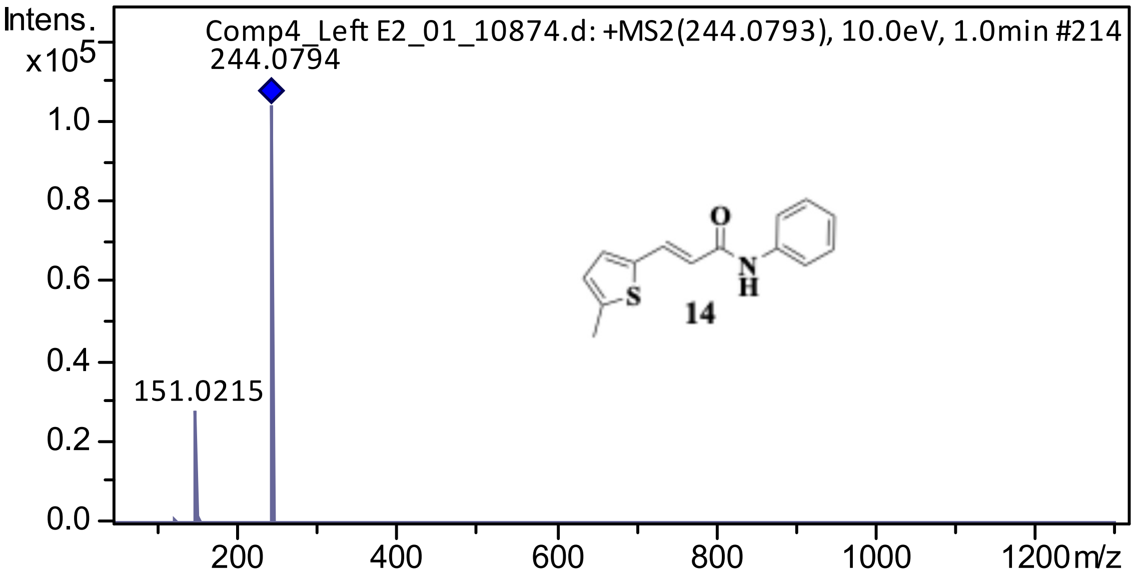

Figure S30. HRMS (QqTOF) m/z spectra **14**.

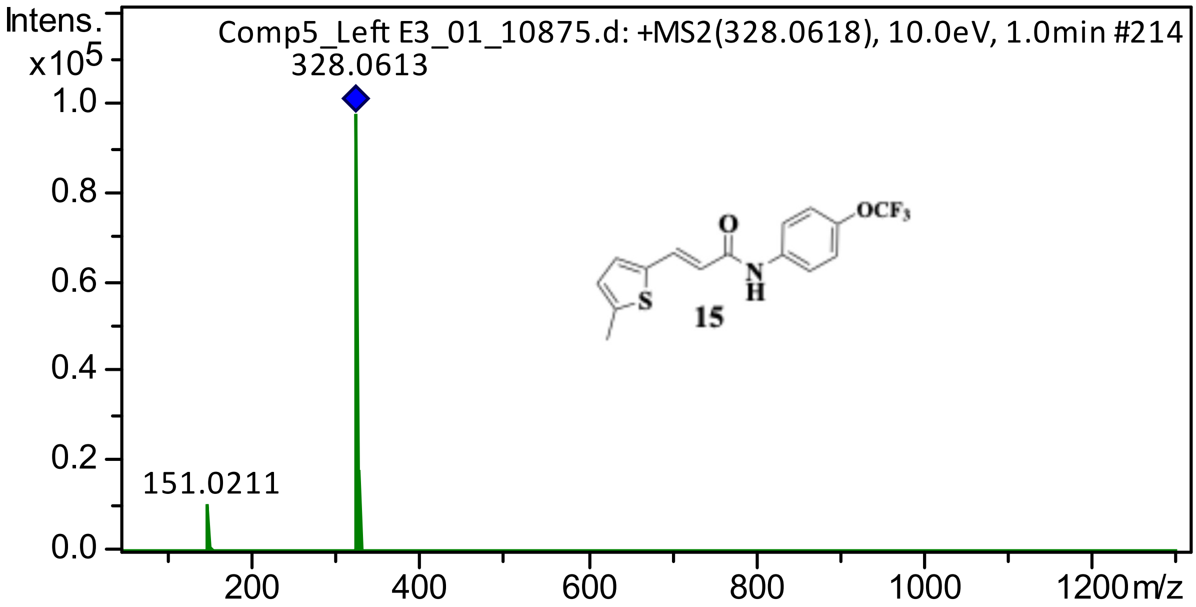

Figure S31. HRMS (QqTOF) m/z spectra **15**.

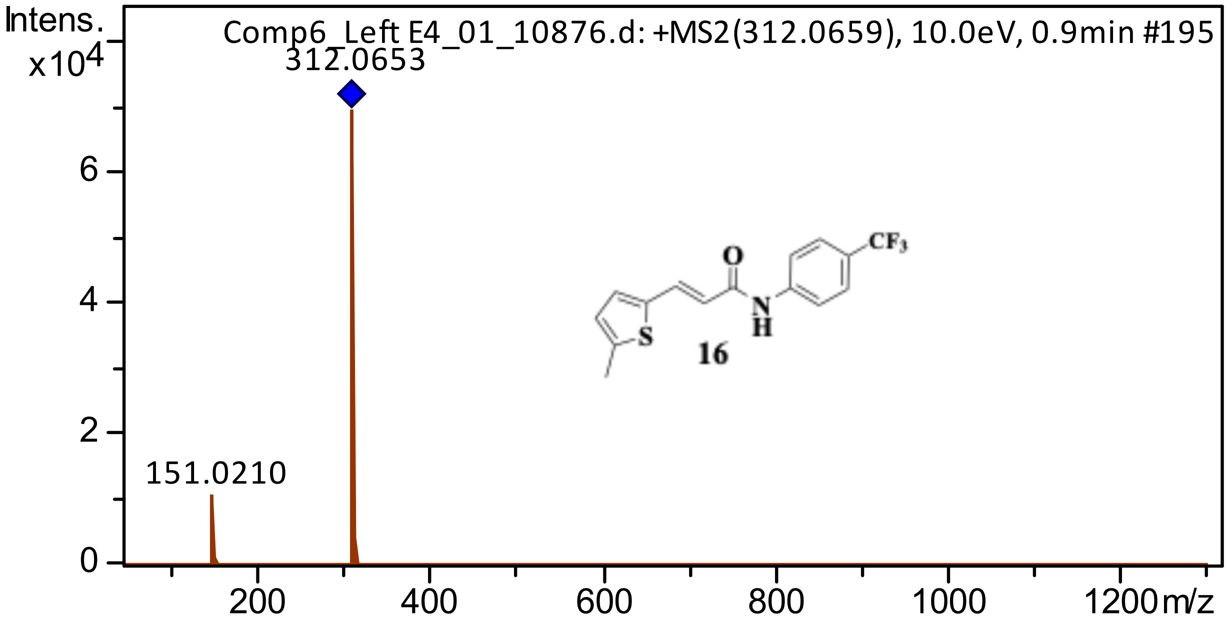

Figure S32. HRMS (QqTOF) m/z spectra **16**.
